# Shotgun metagenomic sequencing reveals the influence of artisanal dairy environments on the microbiomes, quality, and safety of Idiazabal, a raw ewe milk PDO cheese

**DOI:** 10.1186/s40168-024-01980-0

**Published:** 2024-12-20

**Authors:** Gorka Santamarina-García, Min Yap, Fiona Crispie, Gustavo Amores, Cathy Lordan, Mailo Virto, Paul D. Cotter

**Affiliations:** 1 Lactiker Research Group, Department of Biochemistry and Molecular Biology, Faculty of Pharmacy, University of the Basque Country (UPV/EHU), Paseo de La Universidad 7, Vitoria-Gasteiz, 01006 Spain; 2 Bioaraba Health Research Institute-Prevention, Promotion and Health Care, Department of Biochemistry and Molecular Biology, Faculty of Pharmacy, University of the Basque Country (UPV/EHU), Paseo de La Universidad 7, Vitoria-Gasteiz, 01006 Spain; 3 Joint Research Laboratory On Environmental Antibiotic Resistance, Department of Biochemistry and Molecular Biology, Faculty of Pharmacy, University of the Basque Country (UPV/EHU), Paseo de La Universidad 7, Vitoria-Gasteiz, 01006 Spain; 4 Department of Food Biosciences, Teagasc Food Research Centre, Teagasc-The Irish Agriculture and Food Development Authority, Moorepark, Fermoy, Co., Cork, P61 C996 Ireland; 5 APC Microbiome Ireland, University College Cork, Cork, T12 YT57 Ireland; 6 VistaMilk SFI Research Centre, Moorepark, Fermoy, Co., Cork, P61 C996 Ireland

**Keywords:** Metagenomics, Shotgun sequencing, Virulence factors, Antimicrobial resistance genes, Hydrolase-encoding genes, Metagenome-assembled genomes, Cheese quality, Cheese safety

## Abstract

**Background:**

Numerous studies have highlighted the impact of bacterial communities on the quality and safety of raw ewe milk-derived cheeses. Despite reported differences in the microbiota among cheese types and even producers, to the best of our knowledge, no study has comprehensively assessed all potential microbial sources and their contributions to any raw ewe milk-derived cheese, which could suppose great potential for benefits from research in this area. Here, using the Protected Designation of Origin Idiazabal cheese as an example, the impact of the environment and practices of artisanal dairies (including herd feed, teat skin, dairy surfaces, and ingredients) on the microbiomes of the associated raw milk, whey, and derived cheeses was examined through shotgun metagenomic sequencing.

**Results:**

The results revealed diverse microbial ecosystems across sample types, comprising more than 1300 bacterial genera and 3400 species. SourceTracker analysis revealed commercial feed and teat skin as major contributors to the raw milk microbiota (45.6% and 33.5%, respectively), being a source of, for example, *Lactococcus* and *Pantoea*, along with rennet contributing to the composition of whey and cheese (17.4% and 41.0%, respectively), including taxa such as *Streptococcus, Pseudomonas_E* or *Lactobacillus_H*. Functional analysis linked microbial niches to cheese quality- and safety-related metabolic pathways, with brine and food contact surfaces being most relevant, related to genera like *Brevibacterium, Methylobacterium*, or *Halomonas*. With respect to the virulome (virulence-associated gene profile), in addition to whey and cheese, commercial feed and grass were the main reservoirs (related to, e.g., *Brevibacillus_B* or *CAG-196*). Similarly, grass, teat skin, or rennet were the main contributors of antimicrobial resistance genes (e.g., *Bact-11* or *Bacteriodes_B*). In terms of cheese aroma and texture, apart from the microbiome of the cheese itself, brine, grass, and food contact surfaces were key reservoirs for hydrolase-encoding genes, originating from, for example, *Lactococcus, Lactobacillus, Listeria* or *Chromohalobacter*. Furthermore, over 300 metagenomic assembled genomes (MAGs) were generated, including 60 high-quality MAGs, yielding 28 novel putative species from several genera, e.g., *Citricoccus, Corynebacterium*, or *Dietzia*.

**Conclusion:**

This study emphasizes the role of the artisanal dairy environments in determining cheese microbiota and, consequently, quality and safety.

Video Abstract

**Supplementary Information:**

The online version contains supplementary material available at 10.1186/s40168-024-01980-0.

## Background

Dairy products, particularly cheese, are manufactured worldwide using a wide range of production systems and technologies [1]. This leads to a myriad of cheeses with different aromas and textures, making them a versatile and enjoyable product that enriches cultures and, consequently, gastronomic experiences [2]. In particular, raw milk cheeses are regarded as premium dairy products [3], due to their richer and more intense aromatic profiles than pasteurized milk cheeses [4, 5]. Thus, a preference among consumers for raw milk cheeses has been reported [6]. Particular sensory properties of raw milk cheeses are primarily attributed to the microbiota, given their contribution to numerous biochemical reactions that determine cheese quality [7, 8], including lactose metabolism, proteolysis, and lipolysis [8–11]. The microbiota produces a broad range of enzymes (e.g., proteases, lipases, or esterases) and consequent metabolites (e.g., free amino acids, free fatty acids, or volatile compounds), which, together with their subsequent metabolism, determine the aroma and texture of cheese [8, 12]. The microbiota also contributes to ensuring cheese safety, by reducing pathogens through competitive inhibition mechanisms (e.g., bacteriocins or free fatty acids) [10, 13]. Nevertheless, microbial communities can also compromise cheese safety, contributing to the presence of virulence factors (VFs) [14, 15] or antimicrobial resistance genes (ARGs), and thereby serve as potential reservoirs for the transfer to other bacteria, which could include pathogens, in the food chain, including the gastrointestinal tract [16]. Likewise, several undesirable compounds can also be synthetized by the microbiota of cheeses and associated environments [17], such as biogenic amines [10, 18].

Next-generation sequencing (NGS) technologies, such as amplicon (commonly targeting hypervariable regions of 16S rRNA gene) and shotgun sequencing (sequencing of all genetic material), have emerged as indispensable tools for characterizing the microbiota of raw milk cheeses [19–21]. The advantages of NGS for cheese microbiota characterization are the high resolution at the species level, semi-quantitative analysis, and the ability to cover non-cultivable species [21, 22]. Although amplicon sequencing offers cost-effective and rapid profiling of microbial community structure and taxonomy [23], shotgun sequencing facilitates a more comprehensive assessment of the microbiome [21]. This approach not only yields more accurate taxonomic profiling, including the identification of new species [20, 24] but also provides an understanding of the functional potential of microbial communities (e.g., metabolic pathways or genes) [20]. However, using this technology involves increased data complexity, bioinformatics requirements, and associated costs [21].

Overall, the microbiota of raw milk cheeses is composed of a wide range of bacteria, encompassing lactic acid bacteria (LAB), other environmental bacteria, and undesirable bacteria [25]. LAB, including species of several genera, such as *Lactococcus, Lactobacillus, Streptococcus, Enterococcus, Leuconostoc*, or *Carnobacterium* [26], are essential during cheese-making and ripening because of their contribution to the aforementioned biochemical processes that affect cheese flavor and texture, or pathogen inhibition [8, 9, 25, 26]. Nevertheless, in some cases, they have also been related to the production of biogenic amines (BAs) or antimicrobial resistances (AMRs) [10, 18, 27]. The cheese microbiota is also composed of undesirable bacteria, such as particular species of *Staphylococcus, Clostridium*, or *Listeria*, which possess pathogenic or spoilage potential, and other environmental bacteria, which are primarily derived from the cheese production environment [13, 25]. Great differences in the microbial composition among types of cheeses have been reported [28–31] and even among producers of the same type of cheese [25, 32]. Sources of bacteria in milk and cheese are diverse and complex [33], which may include geographical factors (longitude, latitude, or altitude) [34], flock/herd management and feeding [35], lactation stage [33], microorganisms contaminating the teat surface [35], or practices, materials, and ingredients employed during milking or in the dairy environment [26, 36, 37]. Although the impact of the aforementioned factors has been studied, in all cases, the impact of a single or few factors has been analyzed [26, 33, 35–37], none comprehensively studying all or the majority of the potential factors that could have affected. Furthermore, most studies have focused only on cow milk-derived cheeses, with little information relating to cheeses made from the milk of small ruminants, such as sheep [33, 35].

The present work was conducted with the European Protected Designation of Origin (PDO) Idiazabal cheese, a semi-hard or hard cheese manufactured from the raw milk of the autochthonous Latxa and/or Carranzana sheep breeds. It is primarily produced by small-scale artisanal dairies that oversee the entire process, from flock management to cheese-making and sales. The entire production chain, from flock management to milking and cheese-making, occurs in the Southern Basque Country (southwestern Europe), covering an area of 17,213.06 km^2^ (43° 27'–41° 54' N and 1° 5'–3° 37' W). This territory corresponds to the natural habitat of the autochthonous sheep breeds [38]. Flock management involves the use of indoor forage from October to March and semi-extensive or extensive grazing from March to October. Milking and cheese-making processes occur primarily between January and June, following the traditional seasonal approach dictated by the biological rhythms of the sheep [39]. Milking is performed mainly by mechanical systems, and it is refrigerated for subsequent cheese-making. For cheese-making, each producer employs the milk of its own flock and follows the specifications issued by the Idiazabal Designation of Origin Regulatory Board [39]. Briefly, the raw milk collected on the same day is warmed to 25 °C and a starter culture (mixture of *Lactococcus lactis* subsp. *lactis*, *Lactococcus lactis* subsp. *cremoris,* and *Lactococcus lactis* subsp. *lactis* biovar. *diacetylactis*) is added. Milk coagulation occurs at 28–32 °C for 20–45 min, using animal rennet. The resulting curds are cut to a grain size (5–10 mm in diameter), heated to 36–38 °C, molded and pressed for 10–12 h. Then, the cheeses are salted in saturated brine for 24–48 h and finally, ripened in chambers maintained at 80–95% relative humidity and 8–14 °C temperature, at least, for 2 months [39]. Nevertheless, within specifications, producers may adopt various flock management and cheese-making practices that subsequently influence cheese quality and safety, including its sensory attributes. The most significant differences arise from flock management and grazing techniques, such as grazing in valleys or mountains [40], the technological conditions applied during cheese production and ripening [41], and the choice between artisanal (stomachs of Latxa or Carranzana lambs; extracted during the first month of lactation, cleaned, dried, salted and ground) or commercial rennet [42], which has been identified as a key factor shaping the sensory profile of the cheese [43]. At the end of the ripening period, Idiazabal cheese typically measures between 8 and 12 cm in height and weighs between 1 kg and 3.5 kg, containing a minimum of 55% dry extract, with at least 45% fat and a pH range of 4.9 to 5.5. Characterized by its cylindrical shape, the cheese has a hard, smooth rind that varies in color from pale yellow to dark brown in smoked varieties. The paste ranges from ivory white to straw yellow and may have irregular eyes smaller than a grain of rice. In terms of flavor and aroma, it boasts an intense scent that blends dairy, natural rennet, and roasted notes, with a mildly sweet taste, weak to medium acidity, medium saltiness, and no bitterness, resulting in a pronounced persistence and prolonged aroma without unusual sensations [39]. The overall annual amount of Idiazabal cheese produced is approximately 1300 tonnes, with a total of 108 dairies currently, of which only two are large producers. This involves maintaining certain cultural aspects, managing and preserving grazing lands, and supporting the socio-economic development of rural areas [39].

Therefore, the aim of this study was to (1) investigate the microbiota inhabiting the artisanal dairy environments (e.g., herd feed, ingredients, and materials), (2) identify the extent to which these factors influence the microbiota in raw ewe milk and derived cheeses, and (3) explore their functional potential, resistome, virulome and enzymatic potential, in order to unravel the impact of the microbiomes of artisanal dairies on the quality and safety of raw milk cheeses. This study would provide a comprehensive analysis of the microbiomes of artisanal dairy environments and their influence on the quality and safety of raw ewe milk cheeses, offering valuable insights directly applicable to the cheese-making industry.

## Methods

### Sample collection

For sampling, two Idiazabal PDO dairies, identified as A and B, located geographically close to each other (43° 2′ 0.639″–43° 2′ 52.176″ N, 2° 17′ 7.099″–2° 19′ 20.086″ W, Legazpi-Gabiria, Gipuzkoa, Basque Country) were selected to avoid or minimize the impact of geographical conditions (temperature, precipitation, humidity, etc.) on the microbiota [34]. The sampling took place between January and April 2023, with both producers managing flocks consisting of approximately 350–400 Latxa sheep and following similar herd management and cheese-making conditions, as stated before. As indicated in Table 1, several sample types were collected from each producer to identify all the potential microbial reservoirs within artisanal dairies.

**Table 1:** Sampling details (sample type, quantity, and sampling method)

Sample type	Quantity	Sampling method
Feed and forage		
Grass from fresh pastures	250 g	Sterilized samples collection bag (13 × 19 cm) (Deltalab, Barcelona, Spain)
Commercial feed	500 g	
Straw obtained from own fields^a^	150 g	
Homemade feed (blend of grass, corn, beet blend)^a^	500 g	
Teat skin surface	30 swabs (1 swab/10–15 sheep)	Sterile gauze swabs (7.5 cm × 7.5 cm) (Medrull, Dortmund, Deutschland) moistened in 0.9% (w/v) NaCl sterile solution (Scharlab, Barcelona, Spain) and deposited in individual sterile 100 mL bottles (Deltalab, Barcelona, Spain)
Raw milk	1 L	Sterilized 1 L borosilicate glass bottles (Deltalab, Barcelona, Spain)
Dairy surfaces		
Food contact surfaces (e.g., equipment, materials, trays, shelves)	30 swabs	Sterile gauze swabs (7.5 cm × 7.5 cm) (Medrull, Dortmund, Deutschland) moistened in 0.9% (w/v) NaCl sterile solution (Scharlab, Barcelona, Spain) and deposited in individual sterile 100 mL bottles (Deltalab, Barcelona, Spain)
Non-food contact surfaces (e.g., floors, walls)	30 swabs	
Cheese-making ingredients		
Rennet	50 g	Individual sterile 100 mL bottles (Deltalab, Barcelona, Spain)
Brine	1 L	Sterilized 1 L borosilicate glass bottles (Deltalab, Barcelona, Spain)
Dairy samples		
Whey	1 L	Sterilized 1 L borosilicate glass bottles (Deltalab, Barcelona, Spain)
60-day-ripened cheese (1–1.5 kg)	2 units	Sterilized samples collection bag (19 × 38 cm) (Deltalab, Barcelona, Spain)

^a^Straw and homemade feed samples were only collected from Producer A

All the samples were collected aseptically by producers and researchers, using appropriate personal protective equipment, disinfected gloves, and sterile materials to avoid cross-contamination. Samples derived from animals were collected by producers, consequently, the approval of the Ethics Committee for Animal Experimentation was not needed. The samples were transported under refrigeration (3 °C) to the laboratory.

### DNA extraction

Genomic DNA extraction was performed from fresh samples according to the method previously described [25], with some modifications based on previous studies [44–48]. To extract DNA from the cheese samples, 10 g of cheese was suspended in 90 mL of 2% (w/v) sterile sodium citrate (pH 8.0) and homogenized 6 times, each for 20 s on and 10 s off, in a stomacher (Masticator Basic 400, IUL Instruments, Königswinter, Germany). The resulting suspension was centrifuged at 6500 × *g* for 8 min at 4 °C. The supernatant that contained the fat layer was discarded, and the pellet was washed with 50 mL of sodium citrate and centrifuged again (6500 × *g* for 8 min at 4 °C) to harvest the microbiota. The obtained pellet was washed with 800 µL of sodium citrate and centrifuged three times (6500 × *g* for 8 min at 4 °C). For the milk and whey samples, 100 mL were mixed with 200 mL of 2% (w/v) sterile sodium citrate (pH 8.0) and processed as described for the cheese samples without the homogenization step. To extract the DNA from commercial feed samples, 200 g was suspended in 90 mL of buffered peptone water (BPW) and sonicated for 6 min. Then, the commercial feed was removed, and the resulting suspension was centrifuged at 100 × *g* for 1 min at 4 °C to sediment solid impurities that would interfere with sequencing. The resulting suspension was subsequently centrifuged at 15,000 × *g* for 8 min at 4 °C. The supernatant was discarded, and the pellet was washed with 50 mL of BPW and centrifuged again (15,000 × *g* for 8 min at 4 °C). The obtained pellet was washed with 800 µL of BPW and centrifuged thrice (15,000 × *g* for 8 min at 4 °C). The procedure for extracting the DNA from the homemade feed, grass, and straw was the same but the initial starting weights were changed to 200 g, 100 g, and 50 g, respectively. For the teat skin, food contact, and non-food contact surface samples, pools of seven to eight gauzes from each sample type were suspended in 90 mL of BPW and vigorously shaken for 1 min thrice to dislodge microbial communities. Then, the resulting suspension was centrifuged at 100 × *g* for 1 min at 4 °C to sediment solid impurities that would interfere with sequencing, and subsequently processed similarly to the feed samples. For the rennet samples, 10 g of artisanal rennet was suspended in 90 mL of BPW and homogenized 6 times, each for 20 s on and 10 s off, in a stomacher. The resulting suspension was centrifuged at 100 × *g* for 1 min at 4 °C to sediment solid impurities and processed as for the feed samples. For the brine samples, 300 mL of brine was suspended in 600 mL of BPW and it was processed as for the rennet samples but without the homogenization step. The DNA of all the samples was extracted using QIAamp® PowerFecal® Pro DNA Kit (Qiagen, Valencia, CA, USA) following the manufacturer’s instructions, but the elution volume was reduced to 60 µL, and a double elution step was used to increase the DNA yield. Extracted DNA was stored at – 80 °C until sequencing.

The quantity and quality of the extracted DNA were verified by means of a TryCell 2.0 system (Hellma, Southend-on-Sea, UK) coupled to a Cary 50 UV‒Vis spectrophotometer with Varian UV RNA‒DNA estimation application software (version 3.00 (399), Palo Alto, USA). DNA integrity and purity were checked by 1% agarose gel electrophoresis using GelRed dye (Biotium, Inc., Fremont, CA, USA), 10X BlueJuice gel loading buffer (Invitrogen, Waltham, USA), FastGene 100 bp DNA Marker (NIPPON Genetics EUROPE GmbH, Düren, Germany), and the U:Genius 3 system (Synoptics, Cambridge, UK).

### Library construction and shotgun sequencing

Prior to preparing the libraries, accurate DNA quantification was performed in a Qubit® fluorimeter, using Qubit double-stranded DNA (dsDNA) high-sensitivity (HS) and broad-range (BR) assay kits (Bio-Sciences, Dublin, Ireland), following the manufacturer’s instructions. From the extracted DNA, 150 bp paired-end sequencing libraries were prepared for shotgun metagenomic sequencing using the Illumina DNA prep kit (Illumina Inc., San Diego, CA, USA), according to the manufacturer’s instructions and indexed using unique duel indices (UDIs) (Integrated DNA Technologies, Coralville, IA, USA). Following indexing and clean-up, the quantity and quality were checked using a Qubit® fluorimeter and an Agilent 2100 BioAnalyzer system with a high-sensitivity DNA kit (Agilent Technologies, Inc., Santa Clara, USA), respectively. The DNA was pooled equimolarly, a further 0.8 × bead:product clean up was performed with ampure beads (Beckman Coulter), and finally sequenced on a NextSeq 2000 using a P1 300 cycle cartridge, according to manufacturer’s guidelines (Illumina Inc.), at the Teagasc DNA sequencing facility (Moorepark, Cork, Ireland).

### Quality filtering and trimming

All bioinformatic processing was executed with the Teagasc high-performance computing cluster. First, the raw paired-end FASTQ files were trimmed using Cutadapt version 1.18 (https://github.com/marcelm/cutadapt/) to remove adapter sequences. The quality of the reads was assessed using FastQC version 0.11.8 (https://github.com/s-andrews/FastQC), and low-quality reads were removed. The reads were aligned to the ovine genome (*Ovis aries*) using Bowtie2 version 2.4.4 (http://bowtie-bio.sourceforge.net/bowtie2/index.shtml), and all unmapped nonhost reads were assumed to be microbial. Unmapped reads were extracted with samtools version 1.9 (https://github.com/samtools/samtools) and split into paired-end fastq files with bamtofastq from bedtools version 2.27.1 (https://github.com/arq5x/bedtools2).

### Taxonomic classification

Taxonomic classification was performed using Kraken2 (https://github.com/DerrickWood/kraken2), which classifies DNA sequences with high sensitivity and speed by assigning taxonomic labels based on a classification algorithm and exact k-mer matches with reference genomes, against the Genome Taxonomy Database (GTDB) release 89 (https://gtdb.ecogenomic.org/), focusing only on the bacterial reads.

### Diversity analysis

Alpha diversity was calculated in RStudio version 2023.03.1 and R version 4.3.0 (R Core Team, Vienna, Austria, 2023). Data cleaning and preparation for analysis were conducted with the “tidyverse” package (https://github.com/tidyverse). The Shannon, Simpson, Inverse Simpson, Berger, and Shannon evenness (Jevenness and Eevenness) diversity indices were calculated through the “BiodiversityR” package (https://github.com/cran/BiodiversityR), and the Chao1 and ACE diversity indices were calculated with the “vegan” package (https://github.com/vegandevs/vegan). Significant differences between producers for each diversity index were analyzed by means of Kruskal–Wallis analysis in the IBM SPSS statistical package version 26.0 (IBM SPSS Inc., Chicago, IL, USA, 2019). Beta diversity based on Bray–Curtis dissimilarities were also calculated in R through the “vegan” package and plotted into a Principal Coordinate Analysis (PCoA) model based on Bray–Curtis dissimilarities with the “APE” package (https://github.com/cran/ape).

### Source tracker analysis

The sources of the bacterial communities in the raw milk, whey, and cheese samples (sinks) were analyzed by means of Bayesian-based SourceTracker2 version 2.0.1 (https://github.com/caporaso-lab/sourcetracker2). The microbiota of feed, teat skin surface, and food contact and non-food contact surface samples were defined as potential bacterial sources for raw milk, together with rennet for whey, and rennet and brine for cheese. Default settings were selected for the analysis, including a rarefaction depth of 1000, burn-in 100, restart 10, alpha 0.001 and beta 0.01. The percentage influence of each potential bacterial source on each sink was calculated through SourceTracker2.

### Functional potential

The bacterial functional potential of the shotgun metagenomic reads was assessed by means of SUPERFOCUS version 0.34 (https://github.com/metageni/SUPER-FOCUS), using DIAMOND aligner (https://github.com/bbuchfink/diamond) against the SEED database (https://github.com/topics/seed-database), which assigns reads to homologous gene families to determine functional potential. It aggregates these gene families into higher levels of organization to serve a broader function. The highest level of organization in SUPERFOCUS is subsystem level 1, followed by levels 2 and 3, with level 3 representing the most specific functions. The relationship between microbial communities and functional potential was calculated by an orthogonal partial least squares (OPLS) approach applied to log-transformed, when necessary, and UV-scaled data in SIMCA. The microbial communities were selected as X variables, and the functional pathways were selected as Y variables. The model was validated by R2 and Q2 values, Permutation test or Inner Relation plot. The key bacterial communities were identified based on VIP values and loading weights, together with Spearman’s rank correlations calculated in SPSS and interpreted in a heatmap with hierarchical clustering analysis (HCA) performed in R with the “pheatmap” package (https://github.com/raivokolde/pheatmap).

### Resistome analysis

Antimicrobial resistome analysis involving the identification and quantification of antibiotic resistance genes (ARGs) was performed by aligning metagenomic reads using ShortBRED version 1.0 (https://github.com/biobakery/shortbred) against resistance gene markers from the Comprehensive Antibiotic Resistance Database (CARD) (https://card.mcmaster.ca/). The relative abundance of ARGs was expressed as the normalized reads per kilobase per million reads (RPKM) [49]. The relationship between microbial communities and ARGs was calculated by an OPLS approach, as described before.

### Virulome analysis

The identification and quantification of virulence factors (VFs) were performed using ShortBRED (https://github.com/biobakery/shortbred), where shotgun metagenomic reads were mapped against the virulence factor database (VFDB) (http://www.mgc.ac.cn/VFs/main.htm). The relative abundance of VFs was expressed as the normalized reads per kilobase per million reads (RPKM) [49]. The relationship between microbial communities and VFs was calculated by an OPLS approach, as described before.

### Analysis of enzyme-encoding genes

Hydrolase-encoding gene analysis was performed through alignment of reads against ESTerases and alpha/beta-Hydrolase Enzymes and Relatives (ESTHER) database (https://bioweb.supagro.inrae.fr/ESTHER/general?what=index), using DIAMOND version 2.1.7 (https://github.com/bbuchfink/diamond). The best hits were selected for further analysis, and an *e*-value threshold of 10^5^ was established for the mapped contigs. The relationship between microbial communities and hydrolase-encoding genes was calculated by an OPLS approach, as described before.

### Metagenome-assembled genome (MAG) analysis

The metagenome-assembled genomes (MAGs) were assembled using metaSPAdes version 3.13 (https://github.com/ablab/spades). Genome binning was performed with MetaBAT2 version 2.12.1 (https://bitbucket.org/berkeleylab/metabat), with default settings. The quality of the MAGs was checked by means of CheckM version 1.0.18 (https://github.com/Ecogenomics/CheckM). Low-quality MAGs (< 50% completeness and/or > 5% contamination) were removed from downstream analysis, and only medium-quality (50–90% completeness, < 5% contamination) and high-quality (> 90% completeness, < 5% contamination) MAGs were retained for further analysis. Taxonomic classification of MAGs was performed against the Genome Taxonomy Database with GTDB-tk version 2.1.1 (https://github.com/Ecogenomics/GTDBTk).

### Statistical analysis

IBM SPSS statistical package version 26.0 (IBM SPSS Inc., Chicago, IL, USA, 2019) was used for data preparation and analysis. The Mann–Whitney *U* test and Kruskal–Wallis analysis of variance with Bonferroni correction were performed with the SPSS package. The objective was to study differences in the reads and relative abundances of bacterial communities or genetic potential (pathway-related genes, ARGs, VFs, and enzyme-encoding genes), according to the producer and sample type factors. To determine the number of common bacterial genera between the samples, Venn diagrams were generated in R using the “ggvenn” package (https://github.com/yanlinlin82/ggvenn). Permutational Multivariate Analysis of Variance (PERMANOVA) was computed in RStudio version 1.3.959 and R version 3.6.3 by the “vegan” package, to analyze the overall effect of producer and sample type factors on the reads and relative abundance of the bacterial communities or genetic potential. The main bacterial genera and species were selected, their abundances were log-transformed when necessary and UV-scaled, and the corresponding Principal Component Analysis (PCA) was performed and plotted by SIMCA software version 15.0.0.4783 (Umetrics AB, Umeå, Sweden). The number of principal components (PCs) was determined by the eigenvalues (greater than 1.5) and cross-validation. The aim was to study the microbial community distribution according to the producer and sample type. An orthogonal partial least squares discriminant analysis (OPLS-DA) was performed in SIMCA to confirm whether the microbial communities of the samples differed according to the producer and sample type.

## Results

### Characteristics of shotgun metagenomic sequencing data

**Fig. 1:**
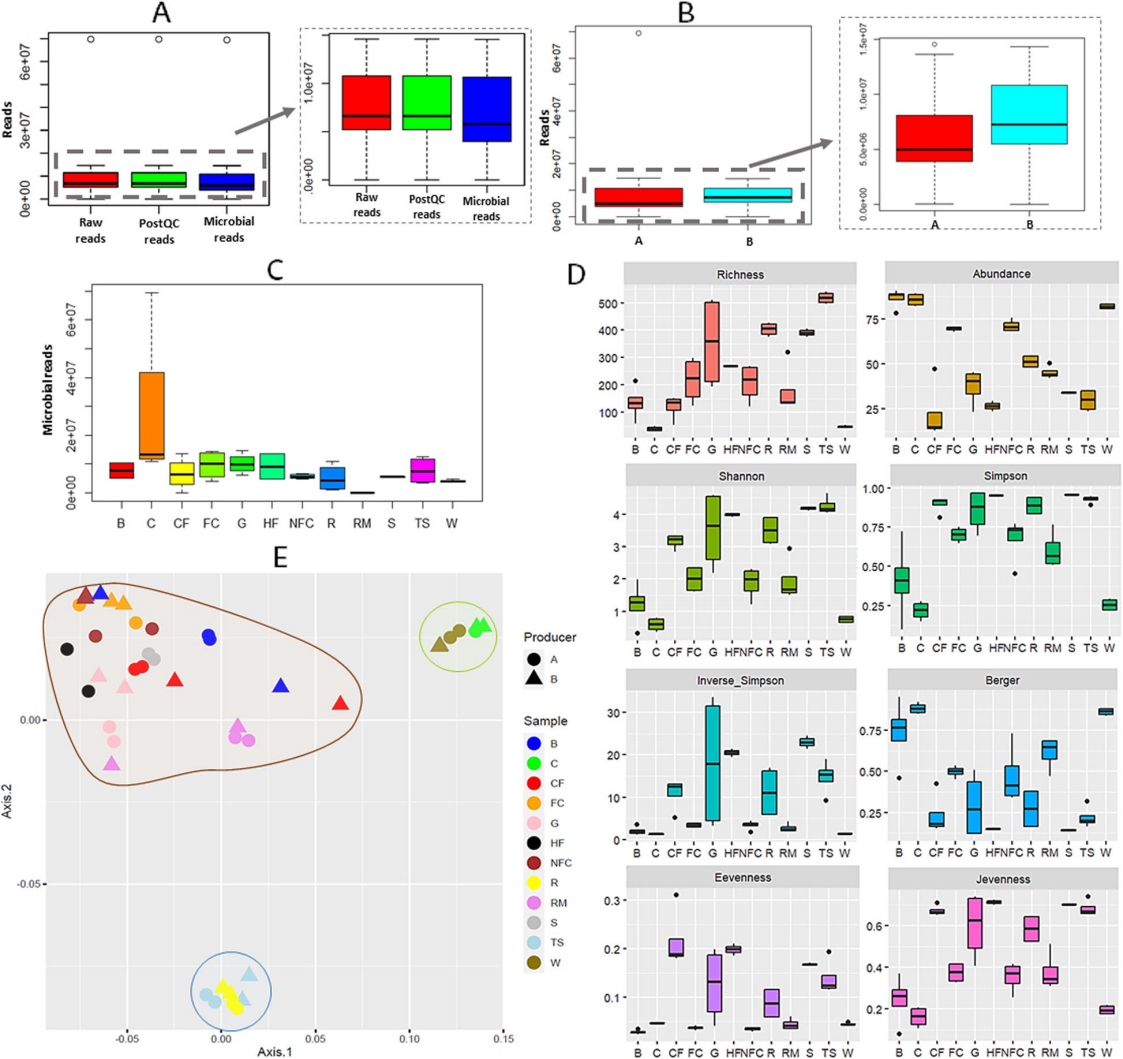
Characteristics of shotgun metagenomic sequencing data, and alpha and beta diversity analyses. Boxplot representation of average raw reads, post-quality control (postQC) reads, and microbial reads obtained from dairy and environmental samples (**A**), boxplot representation of average microbial reads obtained from dairy and environmental samples according to the producer (**B**), boxplot representation of microbial reads according to the sample type (dairy and environmental) (**C**), α-diversity indices calculated for dairy and environmental samples (**D**) and PCoA of β-diversity of dairy and environmental samples (**E**). Abbreviations: B: brine, C: cheese, CF: commercial feed, FC: food contact surfaces, G: grass, HF: home-made feed, NFC: non-food contact surfaces, R: rennet, RM: raw milk, S: straw, TS: teat skin surface, W: whey

Shotgun metagenomic sequencing yielded 71.3 GB of data, with 414,367,387 high-quality paired-end reads and an average of 9,417,440.61 (± 9,993,465.12) reads per sample (Fig. 1A, Supplementary Table S1). Trimming of the raw reads yielded an average of 1553.07 (± 1723.97) reads removed, and 1,156,348.16 (± 1,987,677.59) reads were associated with the ovine (*Ovis aries*) reference genome. The percentage of microbial reads was 82.1% (± 31.3) (Supplementary Table S1). Differences were not observed in the number of microbial reads obtained among the producers (*P* > 0.05) (Fig. 1B). However, the microbial reads differed among sample types (*P* ≤ 0.05), with raw milk samples showing the lowest number (45,179 ± 15,983.59) and cheese samples the greatest (26,775,204.75 ± 28,466,876.45) (Fig. 1C, Supplementary Table S1). Significant differences were also detected in the host reads aligned to the *Ovis aries* genome among sample types (*P* ≤ 0.001), with raw milk, rennet, whey, or teat skin surface samples presenting the highest number of reads (Supplementary Table S1). PERMANOVA confirmed the differences in metagenomic reads among sample types (*P* ≤ 0.001).

### Diversity analyses

In terms of α-diversity (Fig. 1D, Supplementary Table S2), PERMANOVA confirmed clear differences among sample types (*P* ≤ 0.01), but not among producers (*P* > 0.05). Richness indices revealed that the lowest number of bacteria was detected in whey and cheese, followed by commercial feed and brine, whereas artisanal rennet and teat skin surface samples presented the highest richness. Bacterial abundance was greatest in brine, cheese, and whey samples, and lowest in feed (commercial feed, homemade feed, straw, and grass) and teat skin surface samples. Cheese, whey, and brine samples were also the least uniform, indicating that the microbial population was dominated by a few bacteria; whereas feed (straw, homemade feed, commercial feed, and grass), teat skin surface, and artisanal rennet samples reported the greatest uniformity. Considering the number and abundance of bacteria, the greatest biodiversity was observed in the feed (straw, homemade feed, commercial feed, and grass), teat skin swab, and artisanal rennet samples, followed by the raw milk, food-contact, and non-food contact surfaces. Bacteria biodiversity was lowest in the brine, whey, and cheese samples.

In terms of β-diversity (Fig. 1E), PCoA divided the samples into three clusters according to microbial composition and aligned with the α-diversity results. Whey and cheese samples were clustered together since they presented similar bacterial compositions but differed from the rest of the samples. Samples corresponding to the teat skin surface and artisanal rennet also clustered, and the third cluster contained all other samples (raw milk, food contact, and non-food contact swabs and feed samples [straw, homemade feed, commercial feed, and grass]). It should be noted that within the last cluster, greater differences among sample types were observed, indicating greater differences.

### Taxonomic analysis

**Fig. 2:**
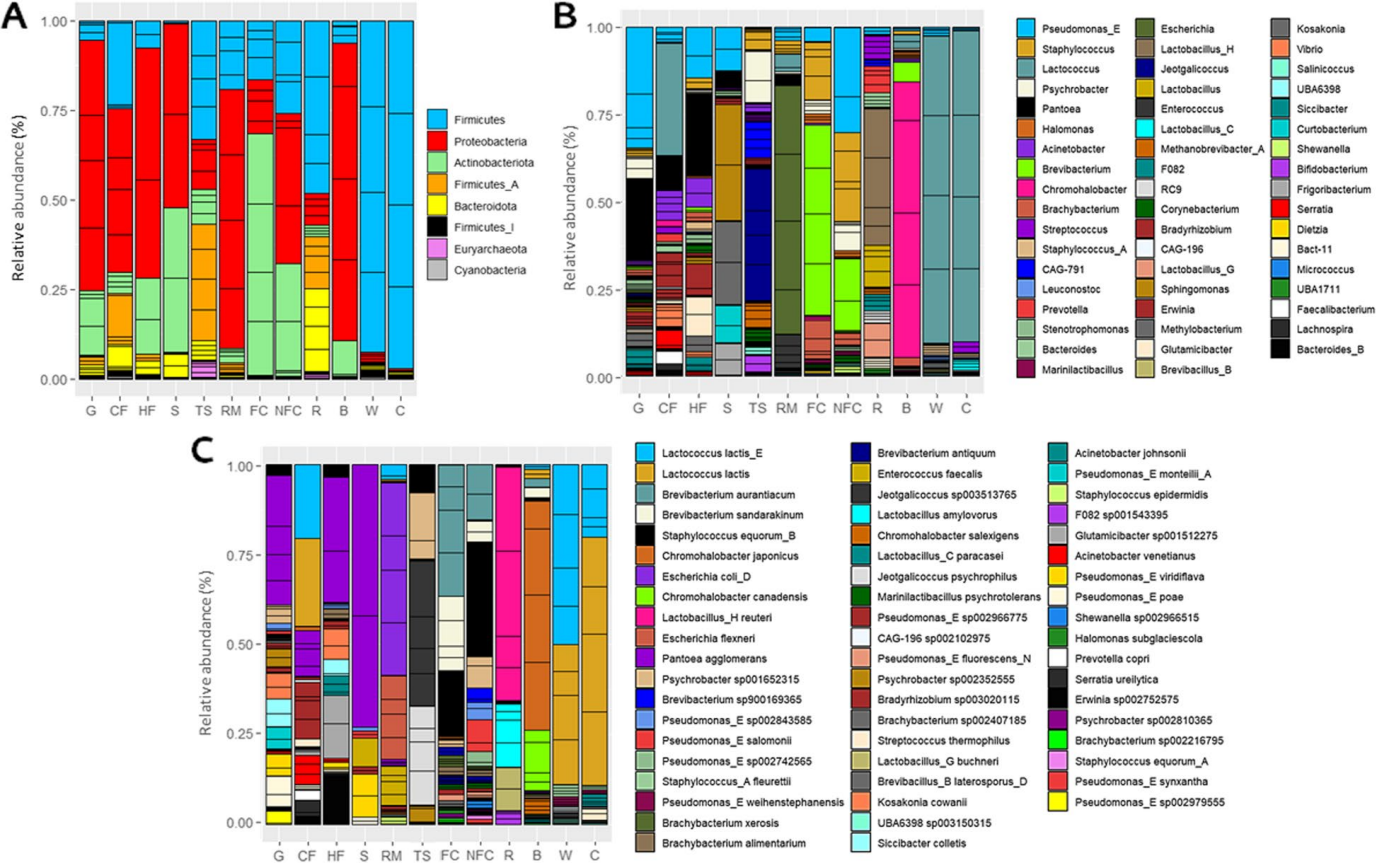
Microbiota of Latxa raw ewe milk, whey, Idiazabal cheese, and environmental samples. Stacked bar chart representation of the taxonomic profiles of the microbiota in dairy and environmental samples at relative abundances greater than 1.00%, at phylum (**A**), genus (**B**) and species (**C**) ranks. Abbreviations: B: brine, C: cheese, CF: commercial feed, FC: food contact surfaces, G: grass, HF: home-made feed, NFC: non-food contact surfaces, R: rennet, RM: raw milk, S: straw, TS: teat skin surface, W: whey

A total of 56 bacterial phyla, 94 classes, 180 orders, 370 families, 1312 genera, and 3467 species were detected among all the samples. An abundance greater than 1% was observed for 8 phyla, of which *Firmicutes, Proteobacteria, Actinobacteriota, Firmicutes_A* and *Bacteroidota* were greater than 5% (Fig. 2A). Similarly, 53 genera were present at average relative abundances greater than 1%, and 14 were present above 5%; namely, *Lactococcus, Staphylococcus, Brevibacterium, Pseudomonas_E, Chromohalobacter, Escherichia, Lactobacillus_H, Psychrobacter, Brachybacterium, Pantoea, Jeotgalicoccus, Lactobacillus, Lactobacillus_G,* and *Streptococcus* (Fig. 2B)*.* In line with the findings for α- and β-diversity, PERMANOVA confirmed the difference in microbiota among the sample types (*P* ≤ 0.01), as indicated by PCA and OPLS-DA analyses (Supplementary Figures S1 and S2). Overall, *Lactococcus* dominated in commercial feed*; Sphingomonas* and *Methylobacterium* in straw*;* and *Pantoea* and *Pseudomonas_E* in grass and homemade feed. Regarding the teat skin surface, *Jeotgalicoccus* and *Psychrobacter* prevailed; *Brevibacterium, Staphylococcus* or *Brachybacterium* were some of the most abundant bacteria on food contact surfaces, while *Pseudomonas_E, Staphylococcus*, *Brevibacterium* or *Psychrobacter* dominated on non-food contact surfaces*.* In the case of raw milk, *Escherichia* dominated, followed by *Enterococcus* and *Lactococcus*. The rennet was mainly composed of *Lactobacillus_H, Lactobacillus, Lactobacillus_G, Prevotella,* and *Streptococcus*, whereas in brine samples, a clear dominance of *Chromohalobacter* was observed, followed by *Brevibacterium* and *Lactococcus*. In whey, *Lactococcus* and *Staphylococcus_A* dominated, and *Lactococcus, Streptococcus,* and *Lactobacillus_C* were the most abundant genera in the cheese (Fig. 2B).

At the species level (Fig. 2C), 58 species had an average relative abundance greater than 1%, and 16 were present above 5%; specifically, *Lactococcus lactis_E, Lactococcus lactis, Brevibacterium aurantiacum, Brevibacterium sandarakinum, Staphylococcus equorum_B, Chromohalobacter japonicus, Escherichia coli_D, Chromohalobacter canadensis, Lactobacillus_H reuteri, Escherichia flexneri, Pantoea agglomerans, Psychrobacter sp001652315, Brevibacterium sp900169365, Pseudomonas_E sp002843585, Pseudomonas_E salomonii* and *Pseudomonas_E sp002742565.* The abundance of the most dominant species also differed among sample types (*P* ≤ 0.01). *L. lactis* dominated in commercial feed, while *P. agglomerans* dominated in the grass and homemade feed. *B. aurantiacum, B. sandarakinum,* and *S. equorum_B* dominated in food contact surfaces, while *S. equorum_B, B. aurantiacum, P. sp001652315, P. salomonii, B. sandarakinum,* and *P. sp002843585* dominated on non-food contact surfaces. *J. sp003513765, J. psychrophilus, and P. sp001652315* dominated in teat skin surface, whereas *E. coli, E. flexneri,* and *E. faecalis* did so in the raw milk. In the rennet, *L. reuteri, L. amylovorus, and L. buchneri* dominated, while *C. japonicus, C. canadensis,* and *C. salexigens* did so in brine. In the whey and cheese samples, *L. lactis_E* and *L. lactis* also dominated, together with other species such as *S. fleurettii* in whey, and *S. thermophilus* and *L. paracasei* in cheese*.* A detailed taxonomic description of the samples can be found in the Supplementary Results.

### Relationship between the environment of artisanal dairies and the microbiota of dairy products

**Fig. 3:**
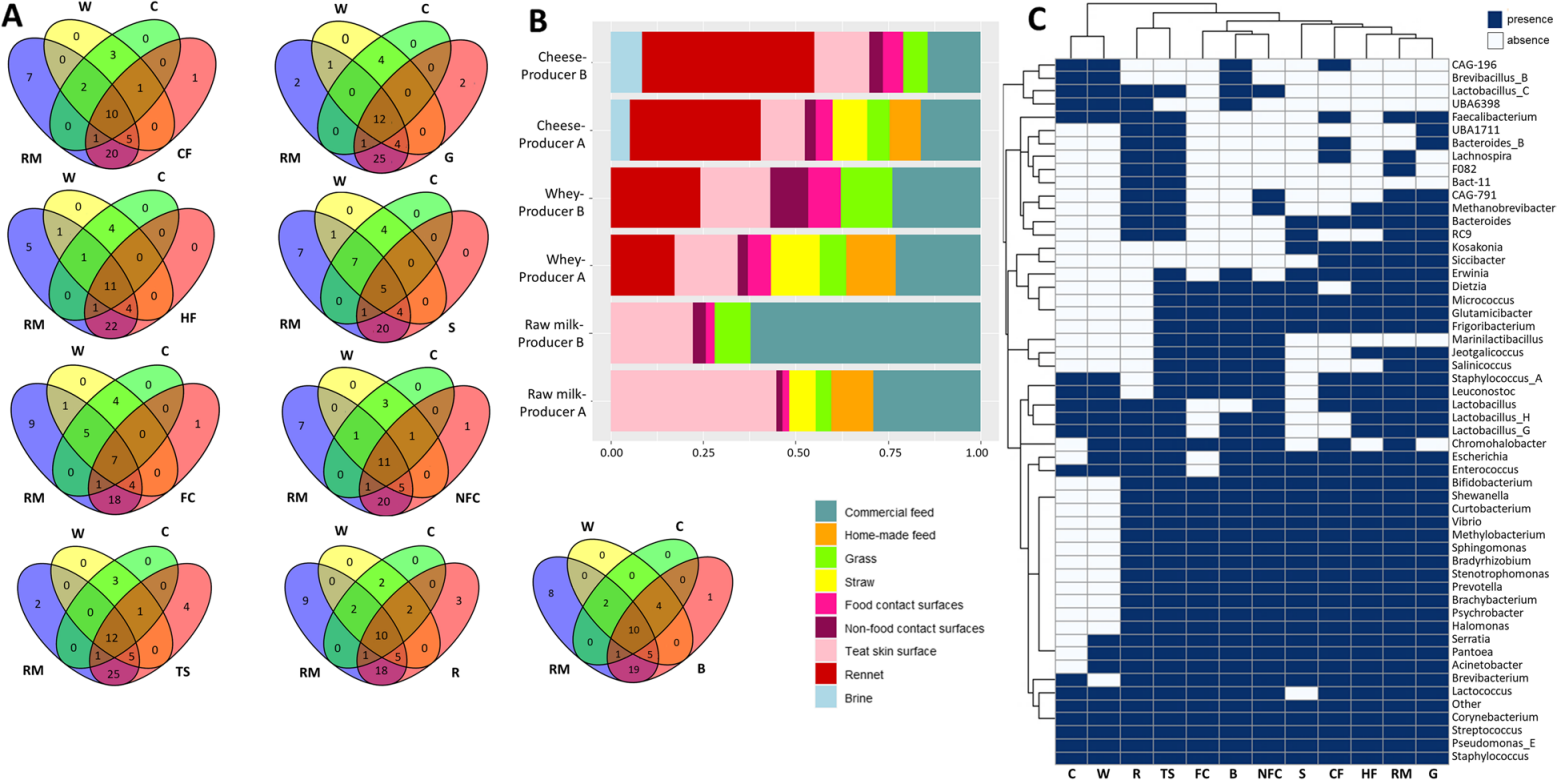
Microbial sources of raw ewe milk, whey, and Idiazabal cheese samples in artisanal dairy environments. Venn diagrams based on the shared bacterial communities among dairy and environmental samples (**A**), stacked bar chart representation of SourceTracker analysis results (unknown proportions are not shown) (**B**), and heatmap representation of the presence or absence of bacterial genera in each of the collected dairy and environmental samples (**C**). Abbreviations: B: brine, C: cheese, CF: commercial feed, FC: food contact surfaces, G: grass, HF: home-made feed, NFC: non-food contact surfaces, R: rennet, RM: raw milk, S: straw, TS: teat skin surface, W: whey

Subsequently, an analysis was done to determine the extent to which microbial communities inhabiting the environment of dairies could contribute to the microbiota of raw milk, whey, and cheese. As shown in Fig. 3A, the Venn diagrams showed that the raw milk, whey, and cheese samples shared many bacterial genera with the environmental samples collected. In the case of raw milk, teat skin surface and grass samples presented the highest number of common bacterial genera (43 and 42, respectively). However, after cheese-making, the brine and teat skin surface samples had the highest number of bacteria in common with whey (19 and 18, respectively) and cheeses (15 and 14, respectively). The food contact surfaces and straw samples exhibited the fewest number of bacteria in common with the dairy samples.

However, overlap at the genus level does not necessarily reflect the origin of the microorganism. Further investigation through SourceTracker revealed commercial feed and the teat skin surface as the main bacterial sources for raw milk (45.6 ± 21.6% and 33.5 ± 14.2%, respectively) (Fig. 3B). More specifically, commercial feed was identified as a likely source of *Lactococcus* and *Pantoea,* together with *Bradyrhizobium* or *Acinetobacter,* among others (Fig. 2B). The teat skin was identified as a source of *Staphylococcus,* as well as *Jeotgalicoccus, Psychrobacter, CAG-791, Methanobrevibacter_A,* or *Bifidobacterium,* for example (Fig. 2B). After cheese production, rennet was reported as the main bacterial source of the microbes found in the cheese (41.0 ± 7.58%). Commercial feed (15.3 ± 2.47%) and the teat skin surface (13.4 ± 2.74%) were also identified as important sources. A similar trend was observed for the origin of whey-associated microbes, even though the contribution of the commercial feed (20.2 ± 4.18%) was greater, followed by rennet (17.4 ± 2.59%) and teat skin (15.4 ± 2.80%) (Fig. 3B). Rennet was identified as a source of *Streptococcus, Pseudomonas_E, Lactobacillus_H, Lactobacillus,* or *Lactobacillus_G,* among others (Fig. 2B). Notably, even if the remaining environmental samples (the rest of the herd feed, food contact and non-food contact surfaces and brine) were, in general, less common sources of milk and cheese microbes, they nonetheless made some notable contributions to the raw milk and cheese microbiota (Fig. 3B, C). For instance, food contact and non-food contact surfaces were a great source of *Brevibacterium, Staphylococcus,* and *Pseudomonas_E* for dairy samples, and *Chromohalobacter* originated, primarily, from the brine (Fig. 2B). The proportion of unknown bacterial sources was low in the raw milk, accounting for 1.24%. This proportion increased considerably in the whey and cheese samples (92.9%) due to the impact of the starter culture used (*Lactococcus lactis*) and its great abundance in whey and cheese (Fig. 2C).

### Functional potential analysis

**Fig. 4:**
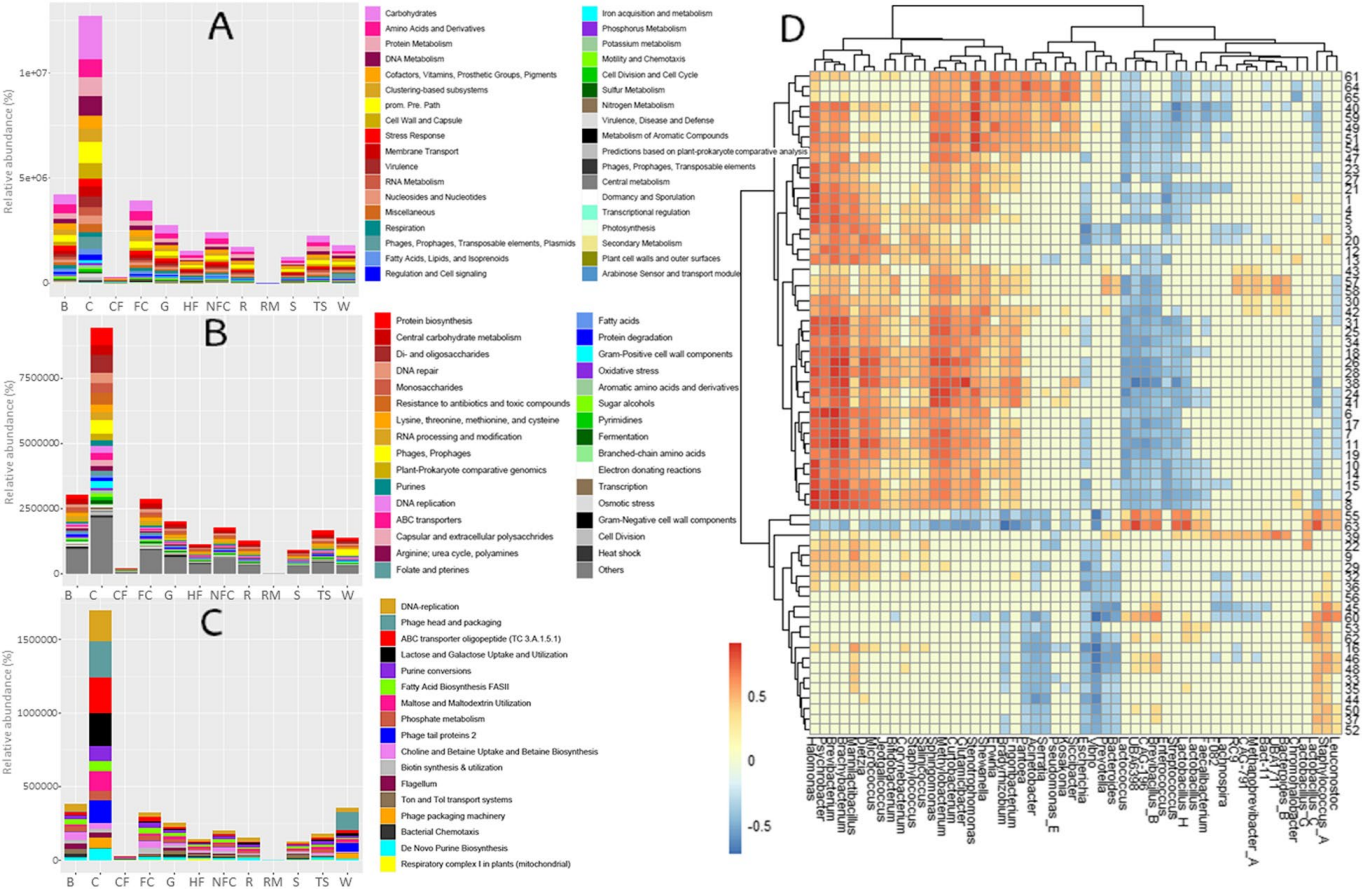
Functional potential of Latxa raw ewe milk, whey, Idiazabal cheese, and environmental samples. Bar chart representation of metabolic pathways at subsystem level 1 (**A**), and main metabolic pathways at subsystem level 2 (**B**) and 3 (**C**), and correlation heatmap between selected food quality and safety metabolic pathways and key bacterial general resulted from OPLS model (**D**). Abbreviations: B: brine, C: cheese, CF: commercial feed, FC: food contact surfaces, G: grass, HF: home-made feed, NFC: non-food contact surfaces, R: rennet, RM: raw milk, S: straw, TS: teat skin surface, W: whey. The numbers on the heat map correspond to abbreviations of the metabolic pathways detailed in Supplementary Table S6

Regarding the functional potential of the microbiomes associated with dairy and environmental samples, a total of 35 functional groups were detected at subsystem level 1, 194 were found at level 2, and 1280 were identified at level 3 (Fig. 4A–C, Supplementary Tables S3–S5). The genes involved in the metabolism of carbohydrates, amino acids and derivatives, and proteins were the most abundant at level 1, followed by DNA, cofactors, vitamins, prosthetic groups and pigments, and clustering-based subsystems metabolisms (Fig. 4A, Supplementary Table S3). At level 2, genes involved in the metabolism of protein biosynthesis, central carbohydrate metabolism, and di- and oligosaccharides, followed by genes involved in DNA repair, resistance to antibiotics and toxic compounds, and monosaccharides metabolism were the most abundant (Fig. 4B, Supplementary Table S4). The most common functional groups at level 3 were DNA-replication, phage head and packaging, ABC transporter oligopeptide (TC 3.A.1.5.1), lactose and galactose uptake and utilization, purine conversions, maltose and maltodextrin utilization or fatty acid biosynthesis (FASII).

Significant differences were observed among samples for the majority of functional groups (*P* ≤ 0.05) (Supplementary Tables S3 and S4), as reflected in the HCA (Supplementary Figures S3–S5). Overall, cheese samples presented the greatest abundance of the most abundant pathways, followed by brine and food contact surfaces (Fig. 4A–C, Supplementary Tables S3–S5). Thus, *Lactobacillus_C, Brevibacillus_B, UBA6398, Staphylococcus_A, CAG-196, Lactobacillus_H, Marinilactibacillus* and *Leuconostoc* were the main genera correlated with the main metabolic pathways.

Specifically, in terms of colonization, various metabolic pathways related to biofilm formation were observed, mainly in non-food contact surfaces, grass, and home-made feed, related to *Brevibacterium, Brachybacterium, Dietzia, Micrococcus* or *Psychrobacter¸* among others. In terms of competitive interactions among bacteria, various metabolic pathways related to bacteriocin production were detected, mainly in cheese, brine, and grass, mainly related to *Sphingomonas, Methylobacterium, Lactobacillus_H, Lactobacillus_C, Brachybacterium, Brevibacterium,* or *UBA6398* (Fig. 4D)*.*

Additionally, various metabolic pathways related to cheese safety were identified. Pathways related to pathogenicity islands and the virulome of pathogens were identified, primarily in cheese, teat skin, and brine (Fig. 4D, Supplementary Tables S5–S7), related to *Halomonas, Brevibacterium, Psychrobacter, Brachybacterium, Sphingomonas, Methylobacterium, Dietzia,* or *Micrococcus*. Pathways related to AMRs were also detected, mainly in cheese, brine, grass, and homemade feed, with the strongest correlations observed for *Stenotrophomonas, Halomonas, Sphingomonas,* and *Methylobacterium,* especially with multidrug, fosfomycin, erythromycin or vancomycin resistance. Pathways related to BAs degradation were identified in cheese, brine, and food-contact surfaces, which were strongly related to *Halomonas, Brevibacterium, Stenotrophomonas, Sphingomonas,* or *Methylobacterium* (Fig. 4D)*.*

Regarding cheese quality, several metabolic pathways related to the metabolism of flavor and texture compounds were identified (Fig. 4D, Supplementary Tables S5–S7). Among others, genes associated with the biosynthesis of amino acids and derivatives (e.g., isoleucine, thiamine, and betaine) were observed, mainly in the grass, food contact surfaces, homemade feed or brine, for which *Halomonas* and *Brevibacterium*, among others, were strongly correlated. Similarly, genes related to catabolism of amino acids (e.g., isoleucine, lysine, or aromatic amino acids) were also detected, mainly in cheese, food contact surfaces, and brine; these genes were also related to *Halomonas* and *Brevibacterium*, among others. Genes involved in the synthesis of fatty acids (FAs) and, especially, polyunsaturated FAs were also detected, mainly in cheese, grass, food contact, and non-food contact surfaces; these genes were strongly correlated with *Brevibacterium, Sphingomonas,* or *Methylobacterium*, among others*.* Carbohydrate metabolism-related genes, such as those involved in the degradation of L-fucose, fructose, and mannose, were also identified, mainly in cheese, brine, and food contact surfaces; these genes were strongly related to *Halomonas* and *Brevibacterium,* among others*.* The metabolism of other compounds, such as alcohols (e.g., mannitol), was mainly observed in cheese, whey, and food contact surfaces, related to *Staphylococcus_A, Erwinia, Leuconostoc, Prevotella, Brevibacillus_B,* or *Lactobacillus_C*. Genes associated with the metabolism of nitrates and nitrites were identified in brine, grass, and food contact surfaces related to *Halomonas, Brevibacterium,* or *Stenotrophomonas*. The metabolism of vitamins (e.g., folate, thiamine, or biotin) was observed in cheese, food contact surfaces, and brine related to *Lactobacillus_C.* Genes associated with terpene metabolism were identified in cheese, food contact, and non-food contact surfaces related to *Sphingomonas, Methylobacterium, Stenotrophomonas, Halomonas, Brachybacterium* or *Psychrobacter,* whereas genes related to the synthesis of carotenoid pigments were identified in food contact, brine and non-food contact surfaces, related to *Brevibacterium*, *Brachybacterium, Halomonas*, and *Methylobacterium,* among others.

Furthermore, genes related to the generation of volatile compounds were identified and related to cheese, food contact surfaces, and brine (Fig. 4D, Supplementary Tables S5–S7), mainly benzoate degradation, sulfur oxidation, alkane synthesis, and to a lesser extent, alkanesulfonate assimilation, toluene degradation or menaquinone biosynthesis, detected mainly in the grass, non-food contact and food contact surfaces. *Halomonas, Brevibacterium, Brachybacterium, Psychrobacter, Sphingomonas, Methylobacterium,* and *Dietzia* were the main bacteria related to these genes*.* Within LAB, *Marinilactibacillus* was correlated with chlorobenzoate degradation, toluene degradation, and butanol biosynthesis; *Leuconostoc* to formaldehyde assimilation and polyprenyl diphosphate biosynthesis; and *Lactobacillus_C* to formaldehyde assimilation and synthesis of aromatic compounds. Moreover, genes related to the synthesis of diverse enzymes, especially aminoglycoside adenylyltransferases or metalloendopeptidases, were also identified and found to be related to *Brevibacterium, Psychrobacter,* or *Halomonas*.

### Resistome analysis

**Fig. 5:**
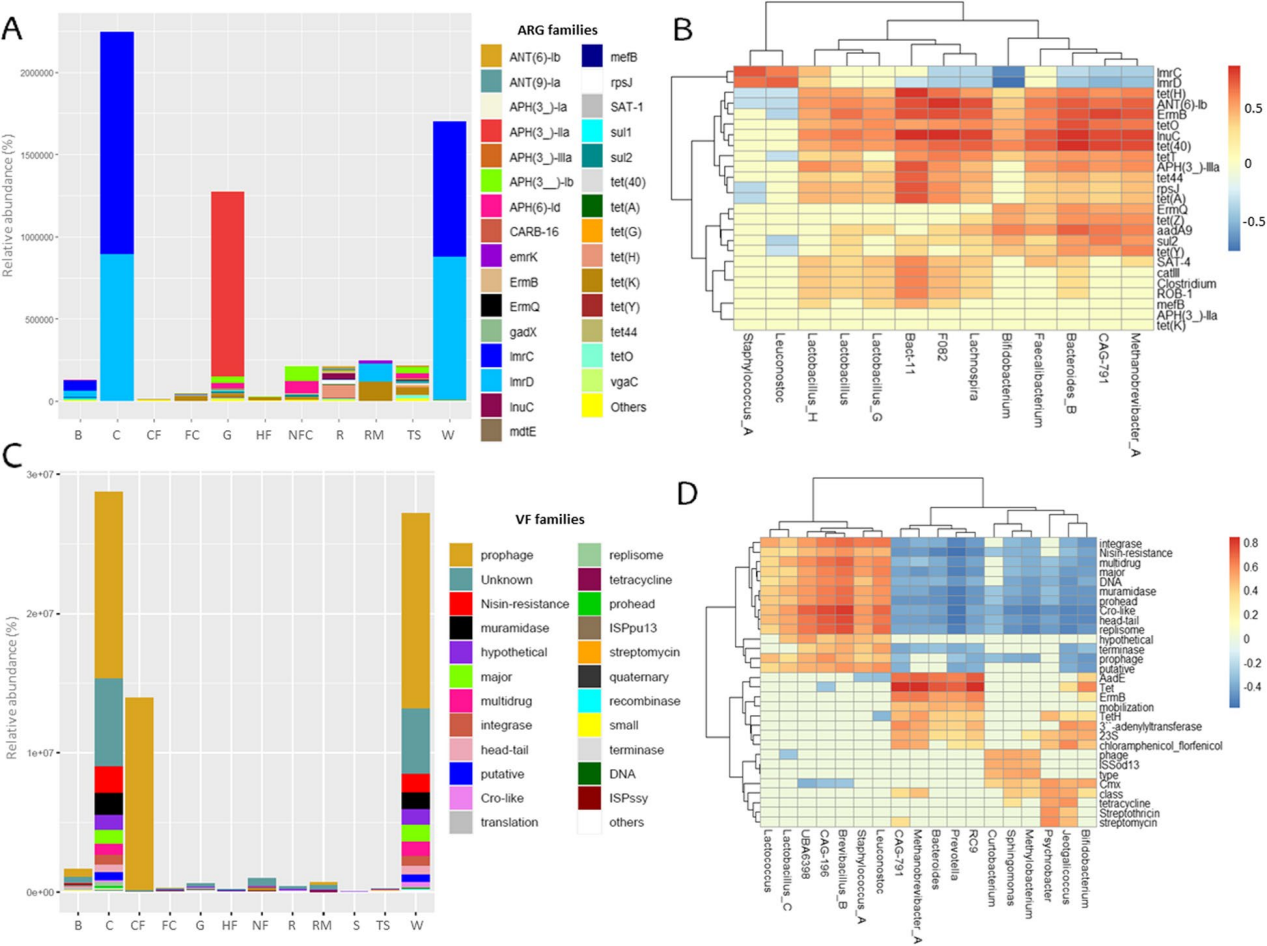
Resistome and virulome of Latxa raw ewe milk, whey, Idiazabal cheese, and environmental samples. Bar chart representation of main ARGs and VFs according to sample type (**A** and **C**), and correlation heatmap between ARGs and VFs, and key bacterial genera resulted from OPLS model (**B** and **D**). Abbreviations: B: brine, C: cheese, CF: commercial feed, FC: food contact surfaces, G: grass, HF: home-made feed, NFC: non-food contact surfaces, R: rennet, RM: raw milk, S: straw, TS: teat skin surface, W: whey

A total of 478 ARGs were detected among all the samples, belonging to 86 ARG families (Supplementary Table S7). ARGs were predicted to confer resistance to antimicrobial peptides and 12 antibiotic classes, i.e., aminoglycosides, fluoroquinolones, fusidanes, glycopeptides, lincosamides, macrolides, nucleoside antibiotics, phenicols, sulfonamides, tetracyclines, β-lactams, and multiple drugs. The highest number of ARG families was related to multidrug (30.2% of ARG families) (primarily against cephamycins, cephalosporins, aminoglycosides, and penicillins), tetracycline (24.4%) and aminoglycoside (18.6%) classes. However, in terms of abundance, lincosamide and aminoglycoside ARG families were the most abundant (total abundance of 4,197,149.04 RPKM and 1,498,605.30 RPKM, respectively) (Supplementary Table S7). Individually, *tet*(K) (accounting for 10.9% of all ARGs), *lmrC* (7.32%), and *lmrD* (6.69%) were the top 3 ARG families detected, whereas, in terms of abundance, *lmrC* (2,245,572.17 RPKM), *lmrD* (1,910,857.10 RPKM) and *APH*(3_)-IIa (1,125,938.83 RPKM) were the most abundant (Fig. 5A, Supplementary Table S7). Additionally, ARGs belong to 6 mechanisms of resistance, namely, efflux, drug inactivation, target protection, target alteration, target replacement, and the combination of efflux and target alteration. Efflux and drug inactivation mechanisms were the most detected (40.7% and 31.4%, respectively) and the most abundant ones (60,489.48 ± 84,275.56 RPKM and 85,679.81 ± 131,558.84 RPKM, respectively).

PERMANOVA confirmed statistically significant differences in the resistome among sample types (*P* ≤ 0.001). The largest number of ARGs were detected on the teat skin surfaces (100 ARGs were detected, with *tet*(K), *sul*2, and *tet*(40) ARG families being the most frequently detected), followed by grass (95 ARGs, with *tet*(K), *tet*(Y) and *sul*2 being the most detected) and rennet (72 ARGs, with *ANT*(6)-Ib and *mef*B being the most detected). Thus, teat skin surfaces, grass, and rennet samples presented the greatest number of ARG families (21, 21, and 19, respectively) (Supplementary Table S7). However, in terms of abundance, cheese presented the greatest abundance of ARG families (2,245,798.91 RPKM), followed by whey (1,701,117.38 RPKM) that were especially related to *lmrC* and *lmrD*, and grass (1,276,130.97 RPKM) due to the abundance of *APH*(3_)-IIa (Fig. 5A, Supplementary Table S7). *lmrC* and *lmrD* were detected in several samples, such as cheese, whey, commercial feed, and brine, whereas *APH*(3_)-IIa was only detected in grass samples (Fig. 5A, Supplementary Table S7).

A total of 13 bacterial genera were strongly related to ARGs, namely, *Staphylococcus_A, Leuconostoc, Bifidobacterium, Bacteroides_B, Lachnospira, Lactobacillus_G, Faecalibacterium, Lactobacillus_H, Lactobacillus, Bact-11, CAG-791, F082,* and *Methanobrevibacter_A* (Fig. 5B). *Staphyloccus_A* and *Leuconostoc* were strongly related to *lmrC* and *lmrD*, while no relationship was observed for *tet*(K) or *APH*(3_)-IIa. For the other ARGs, *tet*(40) was related to *Bacteriodes_B, CAG-791, Methanobrevibacter_A, Faecalibacterium* and *Lachnospira*; *sul*2 to *CAG-791* and, to a lesser extent, *Methanobrevibacter_A* and *Bacteriodes_B*; *tet*(Y) to *Methanobrevibacter_A* and, to a lesser extent, *Bacteriodes_B* and *CAG-791*; and *ANT*(6)-Ib to *F082, Lachnospira, Bact-11, Bacteriodes_B* and *Methanobrevibacter_A*. Overall, *Bact-11* and *Bacteriodes_B,* followed by *CAG-791* and *Methanobrevibacter_A*, were the main bacteria related to the ARGs.

### Virulome analysis

A total of 3193 VFs were detected among all the samples, belonging to 159 VF families (Supplementary Table S8), related to regulatory and genetic elements, metabolism and nucleic acids, enzymes and structural factors, bacterial motility and attachment, transport and secretion, bacteriophages and virus-related factors, antibiotic resistance, molecules and compounds, proteins, toxins and toxin-related genes, and antimicrobials and siderophores classes. VF families were primarily related to enzymes and structural factors (19.5%), followed by antibiotic resistance (13.8%), regulatory and genetic elements (13.2%), and metabolism and nucleic acids (13.2%). Prophage (756 VFs detected), tetracycline (52 VFs), and CP4-6 (36 VFs) were the most common VF families detected. However, in terms of abundance, prophage (total abundance of 42,213,014.74 RPKM), nisin resistance (3,337,734.67 RPKM), and muramidase (2,830,057.90 RPKM) dominated (Fig. 5C, Supplementary Table S8).

Clear differences were observed in the detected VFs and their abundance among sample types (*P* ≤ 0.001), with the highest number of VFs detected in cheese samples (605 VFs), grass (558 VFs), and whey (436 VFs), whereas raw milk, straw, and commercial feed presented the lowest (15, 73, and 140 VFs, respectively). In terms of abundance, the highest abundance of VF families was observed in cheese (total abundance of 28,773,668.41 RPKM), followed by whey (27,237,094.02 RPKM) and commercial feed (13,960,998.20 RPKM), where prophage was the dominant VF family, together with nisin resistance in whey and cheese samples (Fig. 5C, Supplementary Table S8).

A total of 18 bacterial genera were strongly related to VFs (Fig. 5D), namely, *Psychrobacter, Lactococcus, CAG-791, Staphylococcus_A, Leuconostoc, Prevotella, Bacteroides, RC9, Methanobrevibacter_A, Lactobacillus_C, Jeotgalicoccus, CAG-196, Sphingomonas, Methylobacterium, Bifidobacterium, UBA6398, Curtobacterium,* and *Brevibacillus_B*. These bacteria were related to several VF families, especially tetracycline resistance (Tet), aminoglycoside resistance (AadE), Cro-like proteins, head–tail related virulence, replisome, multidrug resistance, integrase, erythromycin resistance (ErmB), major, prohead, and muramidase (Fig. 5D). Specifically, *Brevibacillus_B, CAG-196,* and, to a lesser extent, *Methanobrevibacter_A, RC9, Prevotella, Bacteroides,* and *CAG-791* were among the most related bacteria. Among the dominant VFs, prophages were related mainly to *CAG-196*, and nisin resistance was related to *Brevibacillus_B* and* CAG-196.*

### Enzymatic potential analysis

A total of 17,913,657 genes encoding hydrolases were identified, belonging to 58,593 gene families. The most abundant gene families encode ten types of enzymes, namely, alpha/beta hydrolase abh_upf00227, abhydrolase_5, and abhydrolase_6; bifunctional feruloyl and acetyl xylan esterase (BD-FAE), hydrolase of unknown function (duf_915), lysophospholipase/carboxylesterase, pancreatic lipase, peptidase_S9, peptidase_S15, and proline_iminopeptidase (Supplementary Table S9). Among all the samples, hydrolase-encoding genes from *Lactococcus* (i.e., *L. lactis*) and *Lactobacillus* species (i.e., *L. delbrueckii* subsp*. lactis*) were the most abundant, followed by *Listeria* (i.e., *L. monocytogenes*)*.* The *pepX* gene family from *Lactococcus* species (e.g., *L. lactis* subsp*. lactis*), encoding an Xaa-Pro dipeptidyl aminopeptidase, was the most commonly detected (193,277), followed by cocaine esterases and alpha/beta hydrolases of the family Abhydrolase_5 encoding *YMGC* and *YBCH* gene families from *Lactococcus* (i.e., *L. lactis*) (76,240 and 73,445, respectively).

**Fig. 6:**
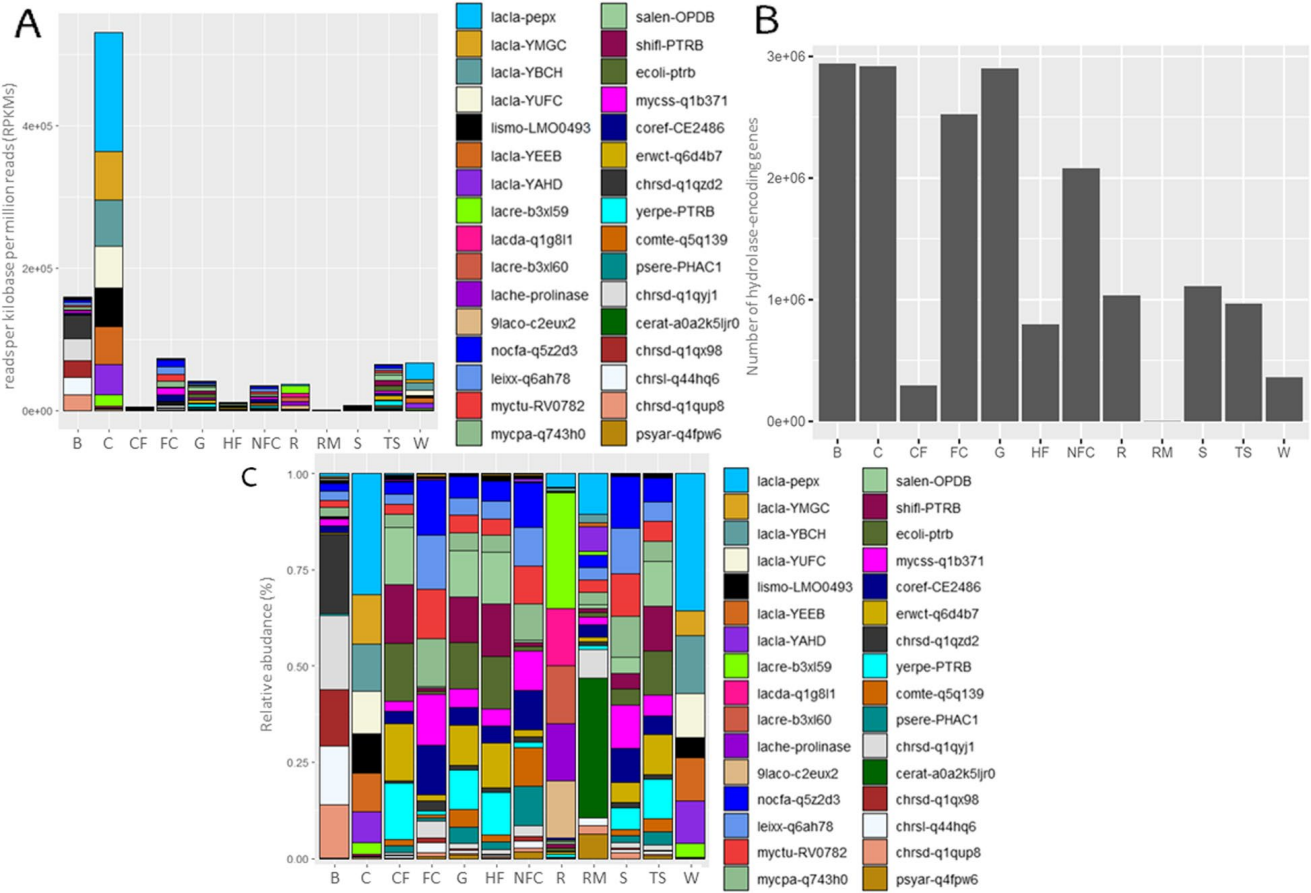
Hydrolase-encoding genes of Latxa raw ewe milk, whey, Idiazabal cheese, and environmental samples. Bar chart representation of main hydrolase-encoding gene families according to sample type (**A**), bar chart representation of the total number of hydrolase-encoding genes according to sample type (**B**), and stacked bar chart representation of main hydrolase-encoding gene families according to sample type (**C**). Abbreviations: B: brine, C: cheese, CF: commercial feed, FC: food contact surfaces, G: grass, HF: home-made feed, NFC: non-food contact surfaces, R: rennet, RM: raw milk, S: straw, TS: teat skin surface, W: whey. Abbreviations of genes encoding hydrolases are detailed in Supplementary Table S9

Clear differences were observed among sample types (*P* ≤ 0.001), with brine presenting the highest number of genes (2,940,987), followed by cheese and grass samples (2,918,313 and 2,898,076, respectively) (Fig. 6B). Thus, the dominant gene families also differed depending on the microbiota of each sample type (Fig. 6C). Protease II-encoding gene families from *Escherichia*, *Shigella,* or *Pectobacterium* and oligopeptidase B-encoding gene families from *Salmonella* or *Yersinia* dominated in the commercial feed, whereas oligopeptidase B-encoding gene families from *Salmonella* and protease II-encoding gene families from *Escherichia* and *Shigella* were notable in the grass, home-made feed and teat skin surfaces. Putative protease-encoding gene families from *Nocardia,* protease II-encoding gene families from *Leifsonia,* or oligopeptidase B-encoding gene families from *Mycobacterium* were found to be dominant on straw and food contact surfaces. In non-food contact surfaces, putative protease-encoding gene families from *Nocardia* or putative protease II-encoding gene families from *Corynebacterium* dominated. Gene families encoding Xaa-Pro dipeptidyl aminopeptidases from *Lactococcus* dominated in the raw milk. Gene families encoding cocaine esterase and oligopeptidase B from *Chromohalobacter* dominated in the brine, whereas Xaa-Pro dipeptidyl aminopeptidase and prolyl aminopeptidase-encoding gene families from *Lactobacillus* dominated in the rennet. Finally, gene families encoding Xaa-Pro dipeptidyl aminopeptidases from *Lactococcus* dominated in whey and cheese.

### Metagenome-assembled genomes (MAGs) analysis

**Fig. 7:**
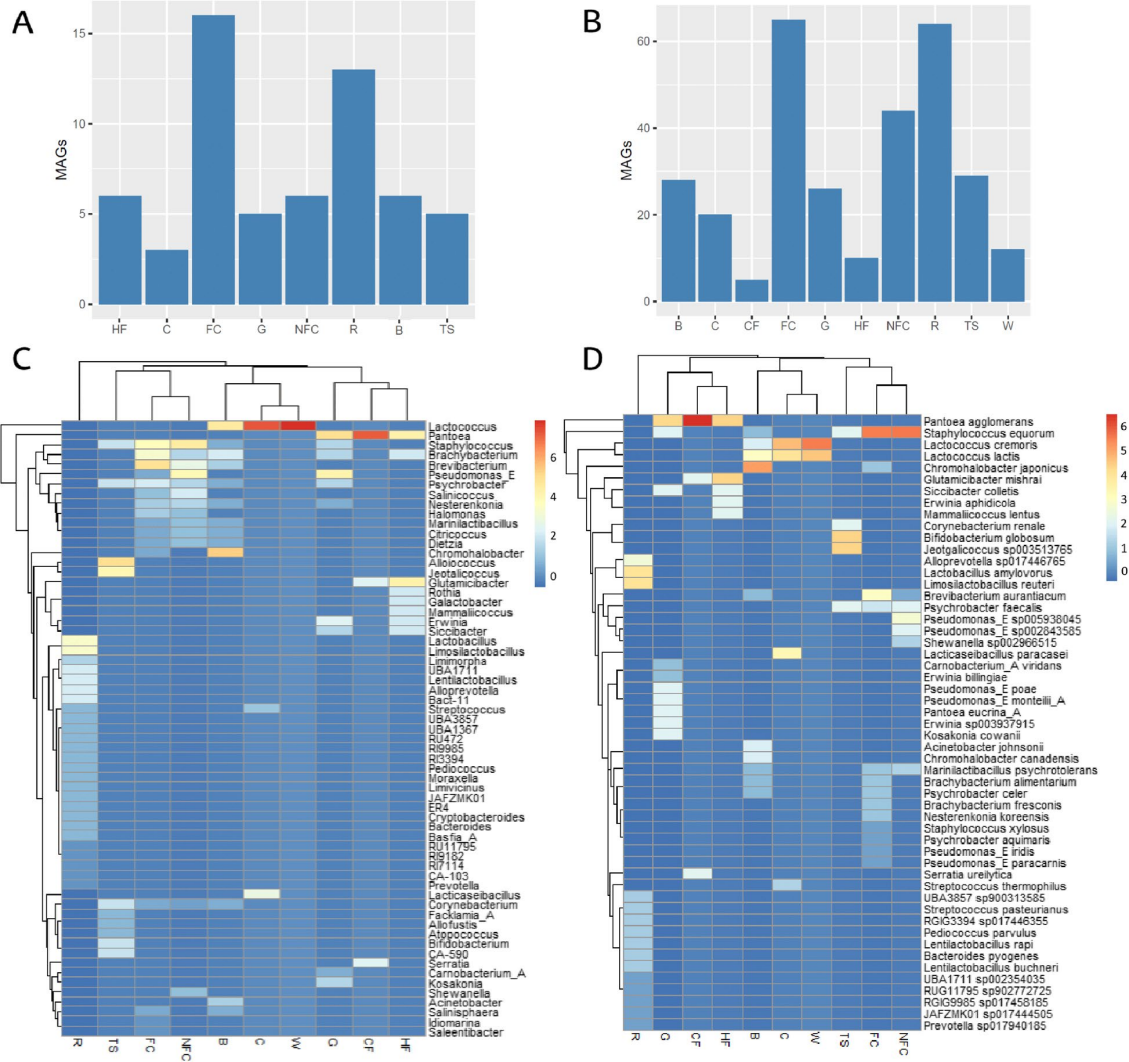
Obtained MAGs from Latxa raw ewe milk, whey, Idiazabal cheese, and environmental samples. Bar chart representation of the obtained high-quality and medium-quality MAGs according to sample type (**A** and **B**, respectively), and heatmap representation of the distribution of MAGs at genus and species taxonomic ranks according to sample type (**C** and **D**, respectively). Abbreviations: B: brine, C: cheese, CF: commercial feed, FC: food contact surfaces, G: grass, HF: home-made feed, NFC: non-food contact surfaces, R: rennet, RM: raw milk, S: straw, TS: teat skin surface, W: whey

A total of 60 high-quality MAGs and 392 medium-quality MAGs were obtained from all the samples (Fig. 7 and Supplementary Figure S6). The MAGs were classified into 63 genera, mainly *Lactococcus* (31 MAGs), *Staphylococcus* (23 MAGs), *Brevibacterium* (20 MAGs), *Brachybacterium* (18 MAGs), *Pseudomonas_E* (14 MAGs), *Psychrobacter* (14 MAGs) and *Pantoea* (11 MAGs). Similarly, MAGs were classified into 53 species, with *Staphylococcus equorum* (21 MAGs), *Lactococcus cremoris* (15 MAGs), *Lactococcus lactis* (14 MAGs), *Pantoea aglomerans* (9 MAGs), *Psychrobacter faecalis* (7 MAGs), *Brevibacterium aurantiacum* (7 MAGs) and *Chromohalobacter japonicus* (7 MAGs) dominating. A total of 123 MAGs could not be classified at the species rank, belonging to 38 genera, primarily *Brevibacterium* (13 MAGs) and *Brachybacterium* (13 MAGs). Moreover, 7 MAGs belonging to the *Carnobacteriaceae* family could not be classified at genera and species rank and 89 MAGs remained unclassified at the phylum rank.

Clear differences were noted in the number of MAGs obtained according to sample type (*P* ≤ 0.001) (Fig. 7A, B, Supplementary Figure S6), with the greatest number of MAGs obtained from food contact surfaces (65 MAGs), rennet (64 MAGs) and non-food contact surfaces (44 MAGs) (Fig. 7B, Supplementary Figure S6C, D). MAGs obtained from food contact surfaces belonged, mainly, to *Staphylococcus, Brachybacterium,* and *Psychrobacter* and, specifically, to *S. equorum* and *B. aurantiacum* species, whereas MAGs from rennet samples belonged to *Lactobacillus,* specifically, *L. amylovorus* and *L. reuteri*. The non-food contact surface MAGs belonged to *Staphylococcus, Pseudomonas_E,* and *Brevibacterium* and, specifically, to *S. equorum* and *P. sp005938045* (Fig. 7C, D and Supplementary Figure S6C, D).

Among the high-quality MAGs, 28 could not be classified at species level, which could correspond to putative new species. These MAGs were collected mainly from food contact surfaces and rennet samples (10 and 6 MAGs, respectively) and, to a lesser extent, from non-food contact surfaces (4), teat skin surfaces (4), brine (3) and homemade feed (1). These MAGs included 2 species from the genus *Basfia_A*, closely related to *Basfia_A succinogenes*; 2 of the genus *Brachybacterium*, related to *Brachybacterium endophyticum* and *Brachybacterium faecium*; 5 *Citricoccus*, related to *Citricoccus muralis* and *Citricoccus zhacaiensis*; 4 *Corynebacterium*, related to *Corynebacterium sp002363255*; 1 *Cryptobacteroides*, related to *Cryptobacteroides sp905234795*; 3 *Dietzia*, related to *Dietzia sp012841845*; 1 *Facklamia_A*, related to *Facklamia_A tabacinasalis*; 1 *Idiomarina*, related to *Idiomarina ramblicola*; 2 *Limimorpha*, related to *Limimorpha sp905234275;* 1 *Moraxella*, related to *Moraxella oblonga*; 1 *Salinicoccus*, related to *Salinicoccus sediminis;* and 2 *Salinisphaera*, related to *Salinisphaera sp002729955*. Moreover, 3 MAGs representative of the family *Carnobacteriaceae* could not be assigned at the genus or species level.

## Discussion

In this study, shotgun metagenomic sequencing was applied to determine to what extent the environment and practices carried out in artisanal dairies could determine the microbiome of raw ewe milk and derived cheeses. The sequencing output of dairy (raw milk, whey, cheese) and environmental samples (homemade feed, commercial feed, straw, grass, teat skin surfaces, food contact surfaces, non-food contact, rennet, and brine) indicated high recovery rates from all sample types, confirming the potential of this methodology to unravel the microbiota of various sample types [24]. Large differences were reported in microbial reads among the sample types, which were confirmed by diversity indices and taxonomic analyses.

Intermediate microbial richness, abundance, and uniformity values were observed in the raw ewe milk, with more than 380 bacterial genera and 450 species, mainly *Escherichia, Enterococcus, Lactococcus, Pseudomonas_E, Staphylococcus,* and *Pantoea*. These findings partially agree with previous analyses of Latxa raw ewe milk [25] and of raw milk collected from other breeds [31, 50, 51], since differences in composition and abundance have been noted. Diversity analyses reported that the whey and cheese samples contained greater bacterial abundance, but lower bacterial richness and uniformity (approximately 60 genera and 80 species), which was related to the great abundance of the starter LAB (SLAB) *L. lactis* and the reduction in the relative abundance of most other bacteria. These findings are partially consistent with previous work on raw ewe milk-derived cheeses [25, 28–30, 52, 53]. Other than SLAB, non-starter LAB (NSLAB), such as *Streptococcus* or *Lactobacillus*, dominated in ripened cheeses, as stated in previous works, although there were differences in the identified NSLAB and their abundance [25, 28, 52, 53]. The presence of undesirable bacteria (e.g., *Staphylococcus_A* and *Pseudomonas_E*) is also in accordance with the findings of previous studies of Idiazabal cheese [25] and other raw ewe milk cheeses, but also at different compositions [28, 32, 53, 54]. Notably, there was a greater abundance of these bacteria in the whey, suggesting greater dissemination of undesirable bacteria in the whey during cheese-making, which has not been previously reported. The presence of SLAB, NSLAB, and undesirable bacteria in whey has also been reported for whey derived from the manufacturing of other raw ewe milk-derived cheeses, although information is scarce [54].

Many of the most abundant bacteria in whey or cheese were not present in the raw milk (e.g., *Lactobacillus_C, Leuconostoc,* or *Staphylococcus_A*), indicating the presence of other potential microbial sources within artisanal dairies [5, 51, 55], as speculated before [25]. With the widely used tool Venn diagram [56] and SourceTracker analysis, which consists of a Bayesian approach model that provides an estimate of the proportion of the community originating from known or unknown source environments [57–59], it was confirmed that all the environmental samples represented notable bacterial reservoirs [5, 25, 51, 55], albeit to different extents. Commercial feed and teat skin surfaces were identified as the main potential sources of bacteria for raw milk, together with the rennet for whey and cheese samples, without taking into account the unavoidable impact of the starter culture, as aforementioned [25, 28–30, 52, 53]. To date, no study has comprehensively assessed all potential microbial sources and their contributions to any raw ewe milk-derived cheese. Individually, the feed used with the herd has been reported to affect the composition and quality of raw milk and derived cheeses [60, 61]. However, metagenomic studies on the feed microbiota and its contribution to the microbial composition of raw milk are scarce [61]. In this study, the analyzed grass, straw, commercial feed, and homemade feed presented intermediate values of microbial richness, abundance, and uniformity, suggesting that these are important reservoirs of bacteria present in the raw milk and cheese (e.g., *Pseudomonas_E, Pantoea,* or *Lactococcus*), which was confirmed by the SourceTracker analysis. Only Tzora et al. [61] have reported that the sheep diet (meal-based diet or flaxseed and lupin-based diet) affects the milk microbiota, including *Corynebacterium* or *Staphylococcus* species*.* Nonetheless, there is little information relating to the presence or absence of the feed bacterial communities in milk [62], and no work has been conducted on cheese.

In the present study, samples from the teat skin surface, together with the artisanal rennet, contained the greatest microbial richness and uniformity. The teat skin surface is considered an important source of raw milk microbes [35, 57, 62, 63], which depends on factors such as animal feed and housing conditions [35]. In previous studies, *Corynebacterium*, *Staphylococcus*, *Moraxella*, *Mannheimia, Jeotgalicoccus,* or *Methanobrevibacter* are reported to be dominant taxa in the teat skin microbiota [35, 63–65]. However, those studies were carried out on cow teat skin [35, 63–65], while information on sheep is scarce [66]. Bi et al. [66] have reported *Bacteroides* and *Prevotella* as dominant genera on the teat skin of ewes, which is not reflected in the present study. Moreover, no study has reported to date if those bacterial communities colonizing the skin surface of sheep are also present in derived raw milk cheeses.

Artisanal rennet, derived from the stomachs of lambs [43, 67], is reported to influence the quality and aroma of cheese through the release of free fatty acids mediated by pregastric lipase [42, 43]. However, the microbial communities of artisanal rennet and their potential impact on cheese have rarely been studied [36]. Hence, only Cruciata et al. [36] have reported through metagenomic techniques that artisanal rennet employed for the production of some raw ewe milk cheeses could be an important source of LAB, such as *Streptococcus* and *Lactobacillus*, even if clear differences are noted in the identified species compared to the present study. Culture-dependent studies of artisanal rennet are also scarce [68–70]. Cosentino and Palmas [68] have reported the presence of coliforms, psychrotrophs, *E. coli* or *S. aureus*, similar to Gil et al. [70] that reported aerobic mesophiles, enterotoxigenic staphylococci and sulphur–reducing *Clostridium*. Notably, apart from LAB, the presence of other major bacterial genera (e.g., *Bacteroides, F082,* or *RC9*) has not been reported to date*.* Likewise, no study has analyzed the transfer of bacterial communities from the rennet to raw milk cheeses.

The rest of the samples, including surfaces in the dairy environment and brine, also contributed to the dairy microbiota, although to a lesser extent. The microbiota of food processing surfaces has been widely studied [71–73], as they are potential microbial niches even after disinfection due to biofilm formation [71]. The microbiota of food processing surfaces has been described as specific to each dairy facility [37]. Several genera and species, such as *Pseudomonas, Psychrobacter,* and *Lactococcus* [71]; *Escherichia, Salmonella,* and *Acinetobacter* [74]; or *Brevibacterium* and *Halomonas* [37], have been described. However, there is no information on dairies producing raw ewe milk-derived cheeses. Similarly, the microbiota of brines is also described as specific to processing facilities [75], with the dominance of LAB and halophilic bacteria (e.g., *Lactococcus, Chromohalobacter, Halomonas* or *Tetragenococcus*) mainly reported within the microbiota of brines used for cheese-making [75–77]. Nevertheless, there is no information on the brines used for the production of raw ewe milk-derived cheeses.

In addition to taxonomic profiling, shotgun metagenomic sequencing enables functional potential analysis of the microbiota [78]. More than 1200 metabolic pathways were detected in this study, primarily related to DNA, carbohydrate, protein, or fatty acid metabolism. Information on the functional potential of the microbiota in raw ewe milk and derived cheeses is scarce [79], and no study has reported the functional contribution of the microbiota related to the dairy environment. Clear differences were observed among sample types, confirming the functional impact of the bacterial communities inhabiting artisanal dairies on cheese quality and safety, as suggested previously [25, 80]. Metabolic pathways related to biofilm formation were identified and related to several bacterial communities inhabiting artisanal dairies, including surfaces (e.g., *Brevibacterium* or *Brachybacterium*), confirming that biofilm formation is one of the potential reasons for the specific communities of dairies [71]. Furthermore, pathways related to competitive inhibition mechanisms, such as bacteriocin production [13], related to bacteria such as *Sphingomonas, Methylobacterium,* or *Lactobacillus_H*, could be the reason for the great abundance of these genera in ripened cheeses or environmental samples [25, 81, 82]. Moreover, several pathways related to cheese safety, such as those associated with pathogenicity or AMRs [80, 83], were identified and related to different dairy and environment samples bacteria (e.g., *Stenotrophomonas, Halomonas,* or *Sphingomonas*), confirming their implication in the safety of raw milk cheeses [25, 80, 83]. Likewise, several pathways related to the metabolism of aroma compounds were identified, related to several genera, such as *Halomonas* and *Brevibacterium,* confirming the impact of environmental and non-desirable bacteria on the aroma of cheese, as suggested previously [9–11, 84].

Several studies have reported the occurrence of ARGs in dairy products [85, 86] or in the dairy environment (e.g., animal feces and soil) [87]. However, information on the contribution of the dairy environment as a source of ARGs is scarce, with tetracycline, aminoglycoside, multidrug, and β-lactam ARGs mainly reported from processing surfaces [71]. Among the more than 470 ARGs detected in this work, multidrug, tetracycline, and aminoglycoside ARG families were the main detected, although lincosamide and aminoglycoside ARG families were the most abundant, partially confirming the functional potential results. Nevertheless, there is no information regarding raw ewe milk and derived cheeses, and the related dairy environment. Grass samples were the main abundant source of ARG families, together with teat skin surface and rennet in terms of a number of ARG families. There is no information on the resistome of any type of teat skin or rennet nor on its contribution to the resistome of milk or cheese. Aminoglycosides, β-lactams, quinolones, tetracyclines, or vancomycin ARG families are reported as most abundant in grass [88, 89], even if there is no information on the grass used for sheep feeding. In dairy surfaces, aminoglycoside, tetracycline, multidrug, and β-lactam ARG families have been detected as dominant [71], but there is no information on the surfaces of dairies producing raw ewe milk-derived cheeses. Similarly, no study has analyzed the resistome of the brine used for cheese-making, with a unique study concerning sea brine, where tetracycline and macrolide ARG families dominate [90]. Similarly, 13 bacterial genera (e.g., *Staphylococcus_A* or *Leuconostoc*) were reported to be primarily related to ARG families in this work. Even if LAB and *Enterobacteriaceae* (e.g., *Staphylococcus* or *Escherichia*) are reported as the main reservoirs of ARG families [1], no study has comprehensively analyzed all the ARG families present and their related hosts in dairy products and related dairy environments by metagenomic techniques. Thus, this work demonstrates the value of sequencing-based methodology for comprehensively identifying potential ARGs reservoirs [78].

Virulence determinants or factors have been widely analyzed in isolates from raw milk or cheese [91, 92]. Here, an exhaustive sequencing-based identification of all VFs present in the microbiome of dairy products and dairy environment is reported for the first time, revealing the potential of this methodology, in terms of food safety, to identify all the VFs present and their relative microbial hosts [78]. More than 3000 VFs were identified, primarily related to prophage or nisin resistance VF families, among others. Phages play a significant role in competitive mechanisms among bacteria and, consequently, in shaping bacterial communities, closely related to the virulence and evolution of numerous critical bacterial pathogens [93, 94]. Phages also affect dairy fermentation by suppressing the growth of lactic acid bacteria through cellular lysis [95]. The presence of genes encoding proteins related to resistance to antimicrobials, primarily nisin and tetracycline, partially agreed with the results for the resistome. Within the environmental samples, grass presented the greatest number of VF families and commercial feed the greatest abundance, indicating potential reservoirs of pathogenic bacteria for raw milk. These results agree with the findings of previous studies of grass microbiota, where several pathogens to humans, for example, *Pseudomonas* species, have been detected [88, 96]. However, there is no information on commercial feed. Moreover, 18 bacterial genera were primarily related to harbor VF families, e.g., *Psychrobacter, Lactococcus, CAG-791,* or *Staphylococcus_A*. For many of these genera pathogenic species, including opportunistic or emerging pathogens, have been previously reported [25].

In terms of cheese quality and flavor, the most noteworthy enzymes related to the metabolism of aroma compounds are hydrolases (EC 3), such as lipases, proteases, or esterases [97]. These enzymes contribute to the most important biochemical processes in terms of aroma development [9], namely, lipolysis and proteolysis [9, 98, 99], with the last also contributing to texture [9]. To elucidate the contribution of the microbiota in this regard, several studies have been published reporting the correlation between microbial communities and cheese-quality compounds [10, 11, 32, 52, 100]. However, the genetic potential of the microbiota in this regard has not yet been studied, this work provides the first exhaustive sequencing-based identification of hydrolase-encoding genes of the microbiomes of dairy (raw ewe milk, whey, and cheese) and environmental samples. Thus, the main hydrolase-encoding genes (e.g., Xaa-Pro dipeptidyl aminopeptidase-encoding gene, i.e., the *pepX* gene) were identified, where *Lactococcus* and *Lactobacillus* were among the main related bacteria. This would confirm the implication of SLAB and NSLAB on aroma development [8–11]. However, hydrolase-encoding genes from other environmental or undesirable bacteria (e.g., *Chromohalobacter* and *Listeria*) were also notable, confirming the potential of these bacteria on cheese aroma [10, 11], and, indeed, the implying impact of the dairy environments [8–11].

Finally, over 300 medium-quality MAGs and 60 high-quality MAGs were generated, 28 of which corresponded to putative novel species, primarily collected from food contact surfaces and rennet samples. Overall, there is little information on the MAGs of raw milk cheeses and dairy environments [79, 101], and information on raw ewe milk-derived cheeses is scarce [79]. The reconstruction of MAGs is evolving into an important tool in food microbiology, due to its capability to identify potential new species and infer their functional potential [102].

## Conclusions

This study shows that artisanal dairy environments, including microbial sources like commercial feed, teat skin, and rennet, play a crucial role in shaping the cheese microbiota. These microbial communities would contribute beneficially to cheese flavor and texture but also pose safety risks through virulence factors and antimicrobial resistance genes. Therefore, these outcomes are of special interest to producers, who could focus on selecting the appropriate feed, enhancing hygiene practices during milking and production, and ensuring surface sanitation. These measures are essential for balancing beneficial and potentially undesirable microbes, while regular microbial monitoring provides further quality assurance, and training staff on safe food handling practices supports overall safety. Additionally, these findings are relevant for regulators, who should establish artisanal-specific guidelines emphasizing microbial control, hygiene standards, and regular inspections to ensure cheese safety. Future studies should delve deeper into the specific factors that shape the microbiota of microbial sources, such as feed or rennet. This includes investigating which types of pasture and feed compositions promote a beneficial microbiota, as well as examining how rennet production parameters influence it. Understanding these influences will enable the optimization of microbial communities for desirable sensory properties and enhanced cheese safety. Practical applications from these insights may include production protocols based on the findings of the study, including the refinement of dairy hygiene protocols to prevent the spread of undesirable bacteria, the implementation of advanced monitoring technologies to track microbiota in real-time, and the development of beneficial microbial inoculants, including biofertilizers, surface inoculants, or starters. Emphasizing these strategies will not only enhance the safety and quality of raw milk cheeses but also support the sustainability and authenticity of artisanal dairy practices, enabling producers to uphold the distinctive traditional qualities of their craft while ensuring product safety, flavor integrity, and consumer confidence.

## Supplementary Information

Additional file 1: Supplementary results. Detailed taxonomic description of the samples.

Additional file 2: Supplementary Table S1. Characteristics of the shotgun sequencing data.

Additional file 3: Supplementary Table S2. Alpha diversity indices.

Additional file 4: Supplementary Table S3. Relative abundances of genes involved in different functional groups at subsystem level 1.

Additional file 5: Supplementary Table S4. Description of data: Relative abundances of genes involved in different functional groups at subsystem level 2.

Additional file 6: Supplementary Table S5. Description of data: Relative abundances of genes involved in different functional groups at subsystem level 3.

Additional file 7: Supplementary Table S6. Description of data: Abbreviations of main metabolic pathways.

Additional file 8: Supplementary Table S7. Relative abundances (RPKM) of antimicrobial resistance gene families.

Additional file 9: Supplementary Table S8. Relative abundances (RPKM) of virulence factor families.

Additional file 10: Supplementary Table S9. Main genes encoding hydrolases, related organisms, and protein names.

Additional file 11: Supplementary Figure S1. Scores and loadings biplot of the PCA model based on the microbiota of dairy and environmental samples.

Additional file 12: Supplementary Figure S2. Scores and loadings biplot of the OPLS-DA model based on the microbiota of dairy and environmental samples.

Additional file 13: Supplementary Figure S3. Heatmap representation of metabolic pathways at subsystem level 1 according to sample type.

Additional file 14: Supplementary Figure S4. Heatmap representation of metabolic pathways at subsystem level 2 according to sample type.

Additional file 15: Supplementary Figure S5. Heatmap representation of metabolic pathways at subsystem level 3 according to sample type.

Additional file 16: Supplementary Figure S6. Bar chart representation of the obtained high quality MAGs at genera (A) and species (B) levels, and medium-quality MAGs at genera (C) and species (D) taxonomic ranks.

## Data Availability

The datasets generated and/or analysed during the current study are available in the European Nucleotide Archive (ENA) repository, https://www.ebi.ac.uk/ena/browser/view/PRJEB73723.
