# Supplementary material for: Shotgun metagenomic sequencing reveals the influence of artisanal dairy environments on the microbiomes, quality, and safety of Idiazabal, a raw ewe milk PDO cheese
Source: Microbiome. 2024 Dec 20;12:262. doi: 10.1186/s40168-024-01980-0 (PMC11662609; doi:10.1186/s40168-024-01980-0)
Supplement: Supplementary file 2 — Additional file 1: Supplementary results. Detailed taxonomic description of the samples. [file 40168_2024_1980_MOESM1_ESM.pdf]

Supplementary results

1-Taxonomic analysis of samples

Regarding the microbiota of each sample, the raw milk was composed of 388 bacterial genera 452 species, within which, 6 genera were above 1%, namely, *Escherichia, Enterococcus, Lactococcus, Pseudomonas_E, Staphylococcus* and *Pantoea.* Moreover, the most important species were *Escherichia coli_D, Escherichia flexneri, Enterococcus faecalis, Lactococcus lactis_E, Staphylococcus epidermidis, Pseudomonas_E sp002966775* and *Pantoea agglomerans.* Along the cheese making process, the microbiota of the generated whey was composed of 71 genera and 84 species, predominating *Lactococcus, Staphylococcus_A, Lactobacillus_C, Pseudomonas_E, Leuconostoc, CAG-196* and *Brevibacillus_B*, being the most important species within this genera *Lactococcus lactis_E, Lactococcus lactis, Staphylococcus_A fleurettii, Pseudomonas_E weihenstephanensis, CAG-196 sp002102975, Brevibacillus_B laterosporus_D, Leuconostoc mesenteroides, Leuconostoc pseudomesenteroides* and *Lactobacillus_H fermentum*. In the case of cheese, 52 genera and 87 species were identified, being the main bacterial genera *Lactococcus, Lactobacillus_C, Staphylococcus_A, Pseudomonas_E, Leuconostoc and CAG-196* and specifically, the species *Lactococcus lactis, Lactococcus lactis_E, Lactobacillus_C paracasei, Staphylococcus_A fleurettii, Pseudomonas_E weihenstephanensis* and *CAG-196 sp002102975*. In general, throughout cheese-making, the majority of predominant bacteria reduced their abundance, except *Lactococcus* in cheese and whey, and *Pseudomonas* in whey. Nevertheless, even reducing their abundance they did not disappear. Likewise, other minority genera (< 0.01%) present in raw milk, such as *Streptococcus, Staphylococcus_A* or *Leuconostoc*, gained importance in whey and cheese. Other genera such as *Lactobacillus_C, Brevibacillus_B, UBA6398, CAG-196* and species such as *Lactobacillus_C paracasei; CAG-196 sp002102975, Staphylococcus_A fleurettii, Pseudomonas_E weihenstephanensis* or *Streptococcus thermophilus* that were not present in raw milk also predominated in whey and cheese (> 1%), which would indicate other potential microbial sources.

In order to identify the microbial sources within artisanal dairies that contribute to the microbiota of milk and also, whey and cheese, different samples were taken in the artisanal dairies. Regarding herd

management and feeding, in the commercial feed 204 bacterial genera and 283 species were identify, predominating *Lactococcus, Bradyrhizobium, Pantoea, Acinetobacter, Vibrio, Serratia, Pseudomonas_E, Faecalibacterium, UBA2912, Erwinia, Prevotella, Paenibacillus_S, Streptococcus, Bacteroides, Bacteroides_B, Chromohalobacter* and *Lachnospira;* and the species *Lactococcus lactis, Lactococcus lactis_E, Bradyrhizobium sp003020115, Pantoea agglomerans, Acinetobacter venetianus, Serratia ureilytica,* and *Prevotella copri.* Other representative species of the dominant genera were *Vibrio furnissii, UBA2912 sp900317095, Erwinia persicina, Streptococcus thermophilus, Bacteroides_B vulgatus, Bacteroides uniformis, Faecalibacterium prausnitzii_D, Pseudomonas_A stutzeri, Chromohalobacter japonicas, Lachnospira eligens_B* and *Paenibacillus_J sp001721045.* In the case of straw, 413 genera and 674 species were identify, although 5 genera predominanted, namely, *Sphingomonas, Methylobacterium, Pseudomonas_E, Curtobacterium* and *Frigoribacterium*, being the most important species within predominating genera *Sphingomonas aerolata, Curtobacterium sp003234355, Frigoribacterium sp001423665, Methylobacterium sp000376345* and *Pseudomonas_E orientalis_A*. In the collected grass, 555 genera and 1030 species were identified, predominating 7 genera*, Pseudomonas_E, Pantoea, Psychrobacter, Siccibacter, Erwinia, Kosakonia* and *Stenotrophomonas*, and the species *Pantoea agglomerans, Pseudomonas_E poae, Siccibacter colletis, Kosakonia cowanii, Pseudomonas_E viridiflava, Pseudomonas_E monteilii_A, Psychrobacter sp002352555* and *Pseudomonas_E sp002979555*. The representative species of the rest of predominating genera were *Erwinia_B gerundensis* and *Stenotrophomonas indicatrix*. Finally, in the home-made feed, 293 genera and 440 species were identified, being the dominant taxa *Pantoea, Pseudomonas_E, Glutamicibacter, Erwinia* and *Acinetobacter,* and predominating Pantoea agglomerans, Glutamicibacter sp001512275 and Erwinia sp002752575. The representative species of the rest of predominating genera were Acinetobacter johnsonii and Pseudomonas_E coleopterorum.

According to the microbiota of the teat skin, 645 genera and 792 species were identified, with 6 bacterial genera predominating (> 1%), namely *Jeotgalicoccus, Psychrobacter, CAG-791, Methanobrevibacter_A, Staphylococcus and Bifidobacterium*; and being *Lactococcus lactis, Lactococcus lactis_E, Lactobacillus_C paracasei,* and *Brevibacillus_B laterosporus_D* the predominant species, and *Staphylococcus_A fleurettii,*

Psychrobacter alimentarius, *Jeotgalicoccus sp003513765* and *Bifidobacterium pseudolongum_A* the most notable species within the aforementioned genera. It should be noted that no species were identified for *Methanobrevibacter_A* genera, despite its' predominance.

In the food contact surfaces of the dairy, 336 genera and 500 species were identified. The predominant genera were *Brevibacterium, Staphylococcus, Brachybacterium, Pseudomonas_E, Psychrobacter, Halomonas, Chromohalobacter, Marinilactibacillus, Dietzia, Corynebacterium, Streptomyces, Micrococcus* and *Acinetobacter*. *Brevibacterium aurantiacum, Brevibacterium sandarakinum, Staphylococcus equorum_B, Brachybacterium xerosis, Brachybacterium alimentarium, Brevibacterium antiquum, Brevibacterium sp900169365, Psychrobacter sp001652315, Brachybacterium sp002407185, Halomonas subglaciescola, Pseudomonas_E fluorescens_N, Pseudomonas_E sp002966775, Marinilactibacillus psychrotolerans, Psychrobacter sp002810365, Brachybacterium sp002216795* and *Chromohalobacter japonicas*. The most representative species of the rest of predominating genera were *Dietzia sp002441635, Corynebacterium glyciniphilum, Streptomyces albidoflavus, Micrococcus sp000224415* and *Acinetobacter albensis.* In the case of non-food contact surfaces, 328 genera and 815 species were identified, with 11 genera dominating, namley *Pseudomonas_E, Staphylococcus, Brevibacterium, Psychrobacter, Brachybacterium, Halomonas, Corynebacterium, Shewanella, Stenotrophomonas, Marinilactibacillus* and *Salinicoccus*. At species level, dominated *Staphylococcus equorum_B, Brevibacterium aurantiacum, Psychrobacter sp001652315, Pseudomonas_E salomonii, Brevibacterium sandarakinum, Pseudomonas_E sp002843585, Pseudomonas_E sp002742565, Brevibacterium sp900169365, Shewanella sp002966515, Brachybacterium alimentarium, Brevibacterium antiquum, Pseudomonas_E fluorescens_N, Marinilactibacillus psychrotolerans, Staphylococcus equorum_A, Pseudomonas_E synxantha* and *Pseudomonas_E sp002966775*. The most representative species of the rest of predominating genera were *Halomonas subglaciescola, Corynebacterium glyciniphilum, Stenotrophomonas sp003484865 and Salinicoccus halodurans*.

In the artisanal rennet, 484 bacterial genera and 850 species were identified, within which, 13 bacterial genera were dominant (> 1%), namely, *Lactobacillus_H, Lactobacillus, Lactobacillus_G, Prevotella, Streptococcus, F082, Bacteroides, RC9, Bact-11, UBA1711, Pseudomonas_E, CAG-791* and

Methanobrevibacter_A. The dominant species *were Lactobacillus_H reuteri, Lactobacillus amylovorus, Lactobacillus_G buchneri* and *F082 sp001543395*. The most representative species of the rest of predominating genera were *Prevotella sp002393725, Streptococcus pasteurianus, Bacteroides pyogenes, RC9 sp900319685, Bact-11 sp002361615, UBA1711 sp002354035, Pseudomonas_E fragi_B, CAG-791 sp002394525* and *Methanobrevibacter_A sp900314615*.

Finally, the brine used for cheese-making contained 273 bacterial genera and 432 species, within which, *Chromohalobacter, Brevibacterium, Lactococcus, Brachybacterium, Halomonas, Staphylococcus*, *Acinetobacter*, *Pseudomonas_E, Stenotrophomonas, Psychrobacter* and *Marinilactibacillus* dominated, and the species *Chromohalobacter japonicus, Chromohalobacter canadensis, Chromohalobacter salexigens, Brevibacterium sandarakinum, Lactococcus lactis, Brevibacterium aurantiacum, Lactococcus lactis_E, Staphylococcus equorum_B, Acinetobacter johnsonii, Brachybacterium alimentarium,* and *Brachybacterium sp002407185*. The most representative species of the rest of predominating genera were *Halomonas shengliensis, Pseudomonas_E azotoformans_A, Stenotrophomonas lactitubi* and *Marinilactibacillus psychrotolerans.* Overall, as shown in Figure XB, it was confirmed that predominant bacteria of raw milk, whey and cheese were also present in the environmental samples of artisanal dairies.
