# Supplementary figures and images for: Shotgun metagenomic sequencing reveals the influence of artisanal dairy environments on the microbiomes, quality, and safety of Idiazabal, a raw ewe milk PDO cheese
Source: Microbiome. 2024 Dec 20;12:262. doi: 10.1186/s40168-024-01980-0 (PMC11662609; doi:10.1186/s40168-024-01980-0)

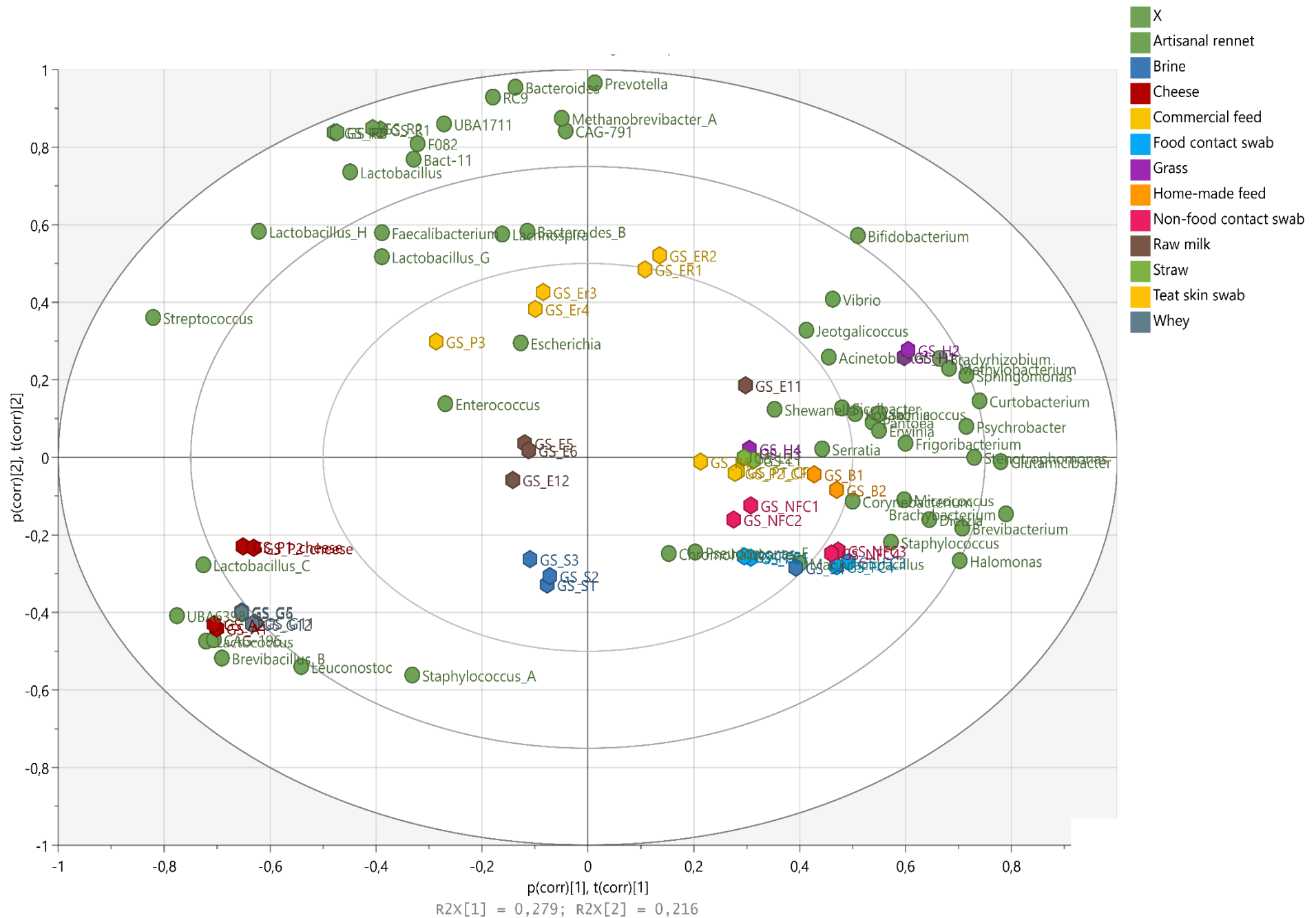

Supplement: Supplementary file 12 — Additional file 11: Supplementary Figure S1. Scores and loadings biplot of the PCA model based on the microbiota of dairy and environmental samples. [file 40168_2024_1980_MOESM11_ESM.pdf]

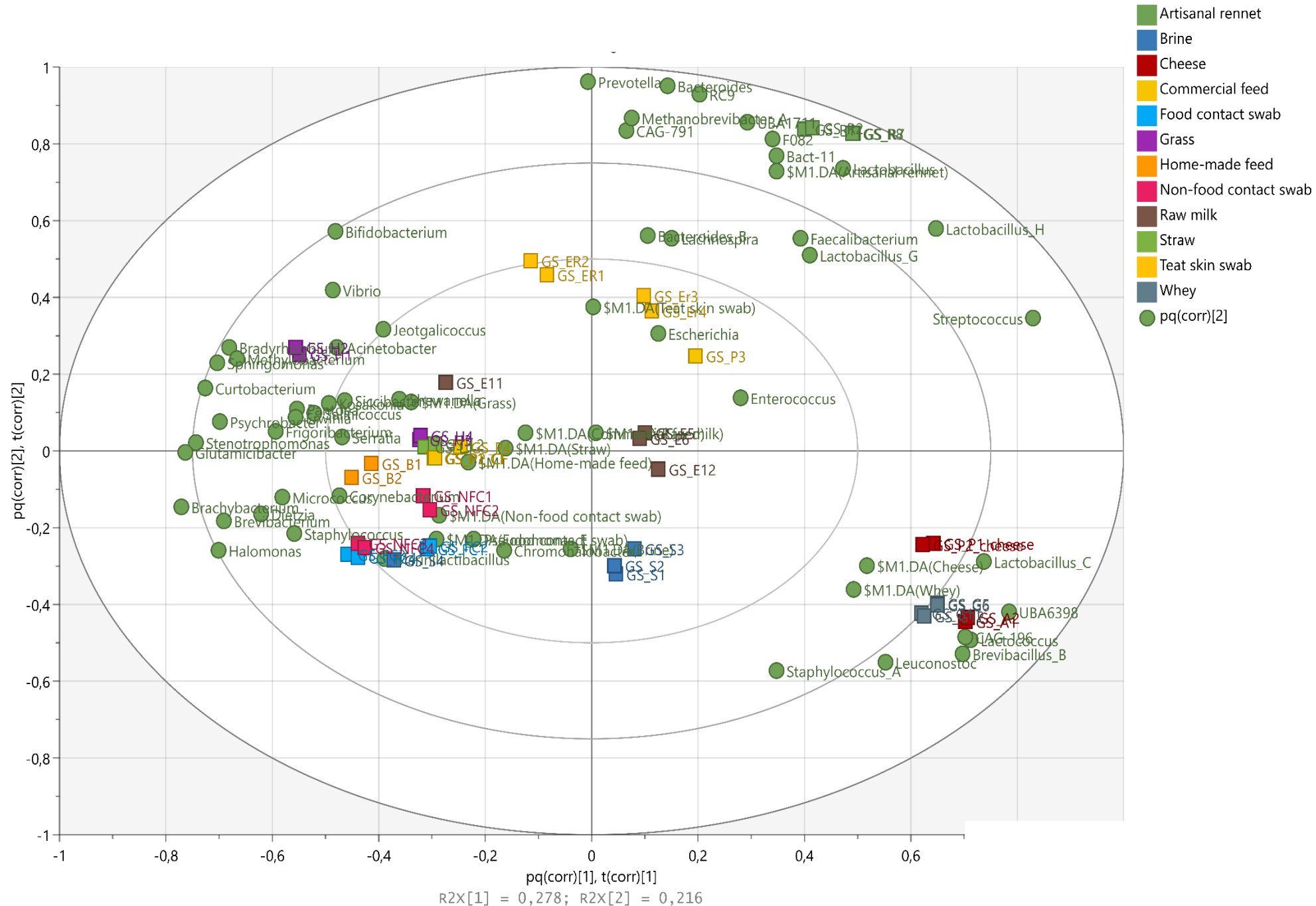

Supplement: Supplementary file 13 — Additional file 12: Supplementary Figure S2. Scores and loadings biplot of the OPLS-DA model based on the microbiota of dairy and environmental samples. [file 40168_2024_1980_MOESM12_ESM.pdf]

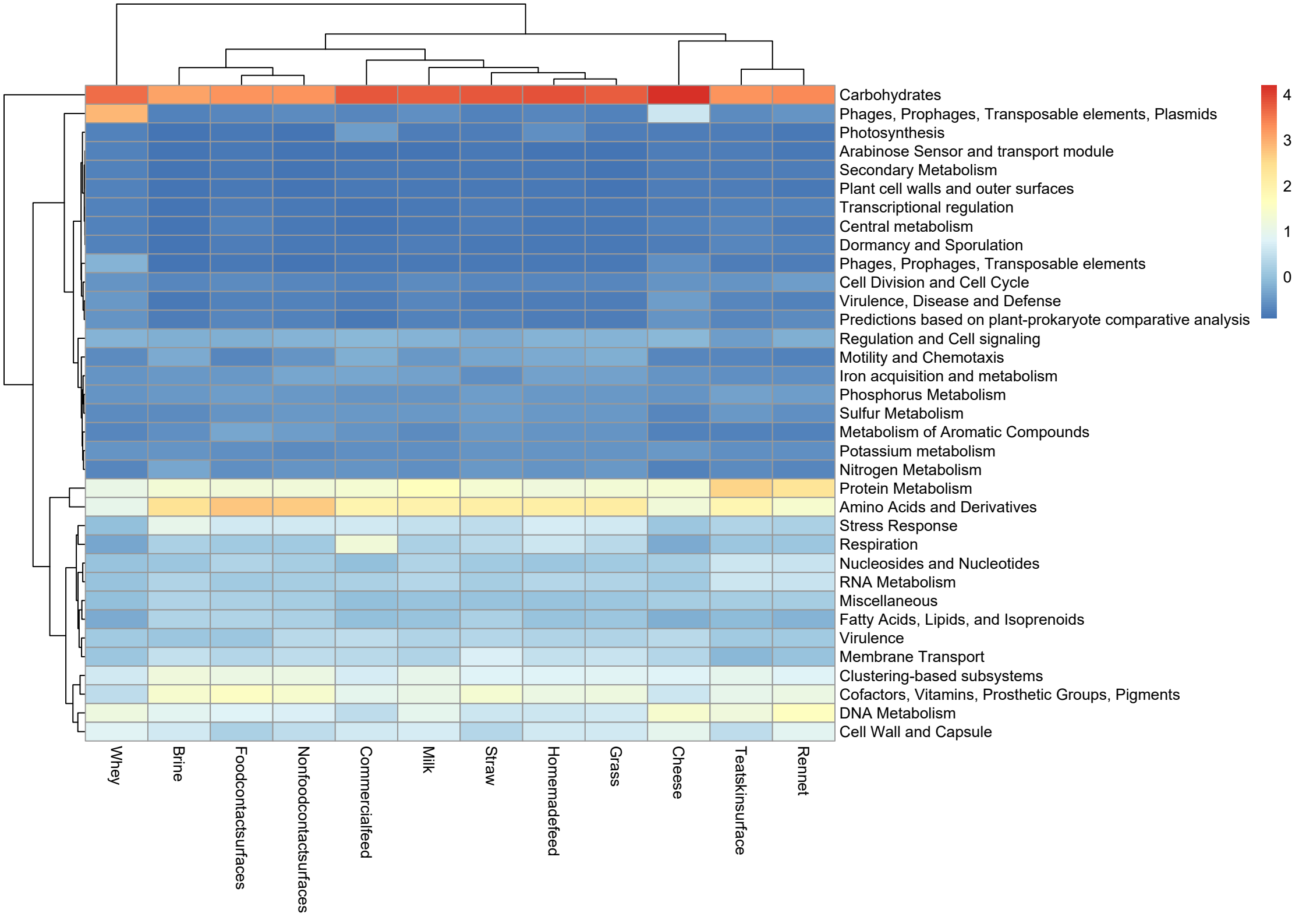

Supplement: Supplementary file 14 — Additional file 13: Supplementary Figure S3. Heatmap representation of metabolic pathways at subsystem level 1 according to sample type. [file 40168_2024_1980_MOESM13_ESM.pdf]

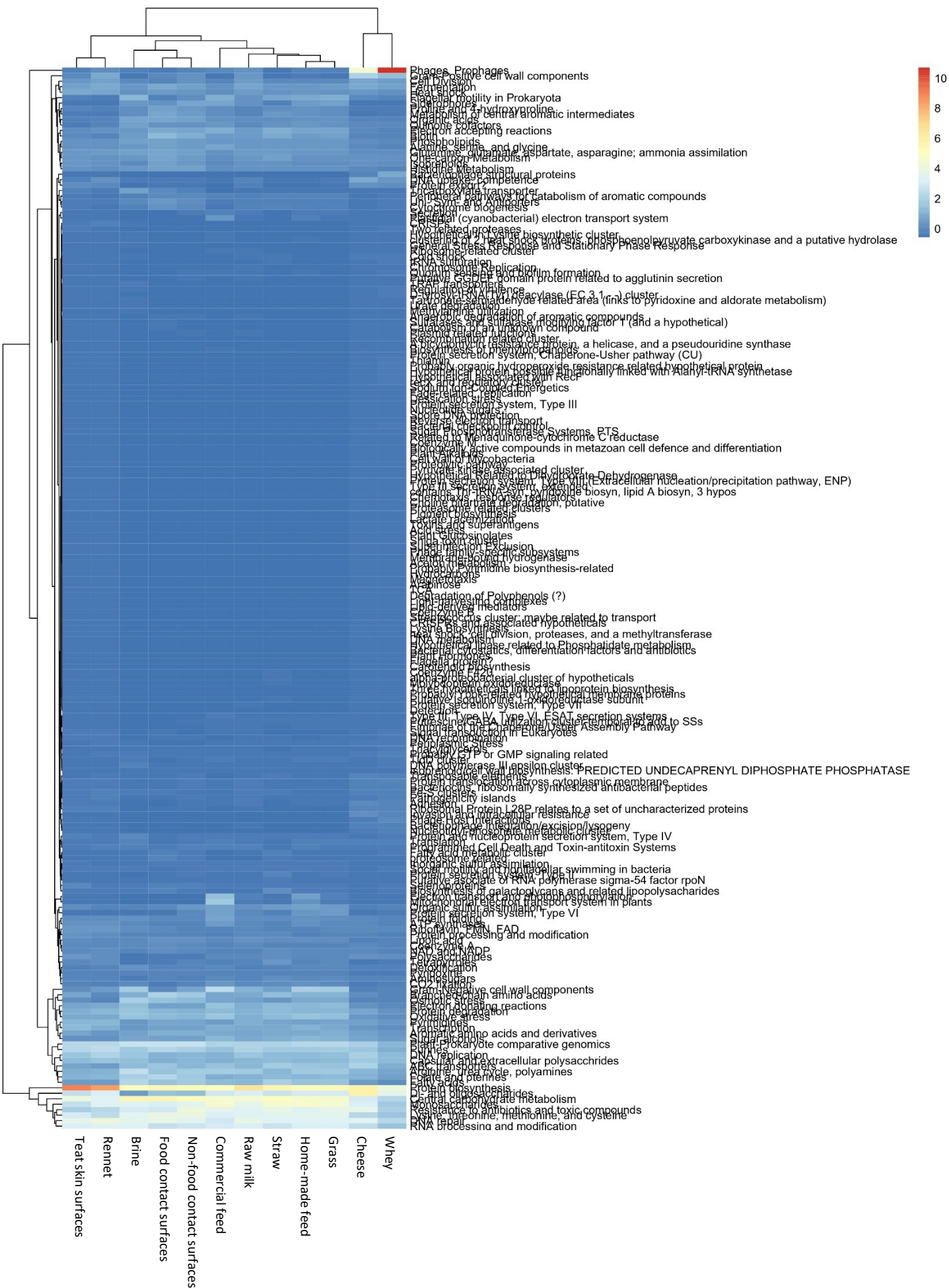

Supplement: Supplementary file 15 — Additional file 14: Supplementary Figure S4. Heatmap representation of metabolic pathways at subsystem level 2 according to sample type. [file 40168_2024_1980_MOESM14_ESM.pdf]

**A**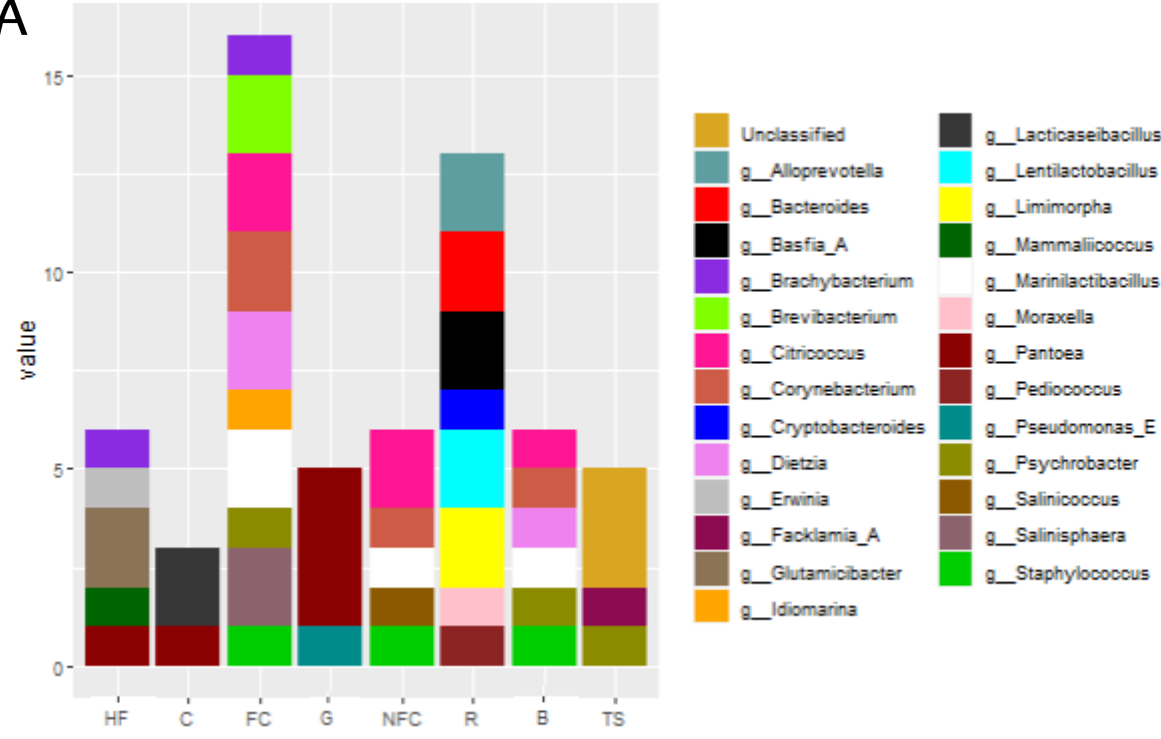**B**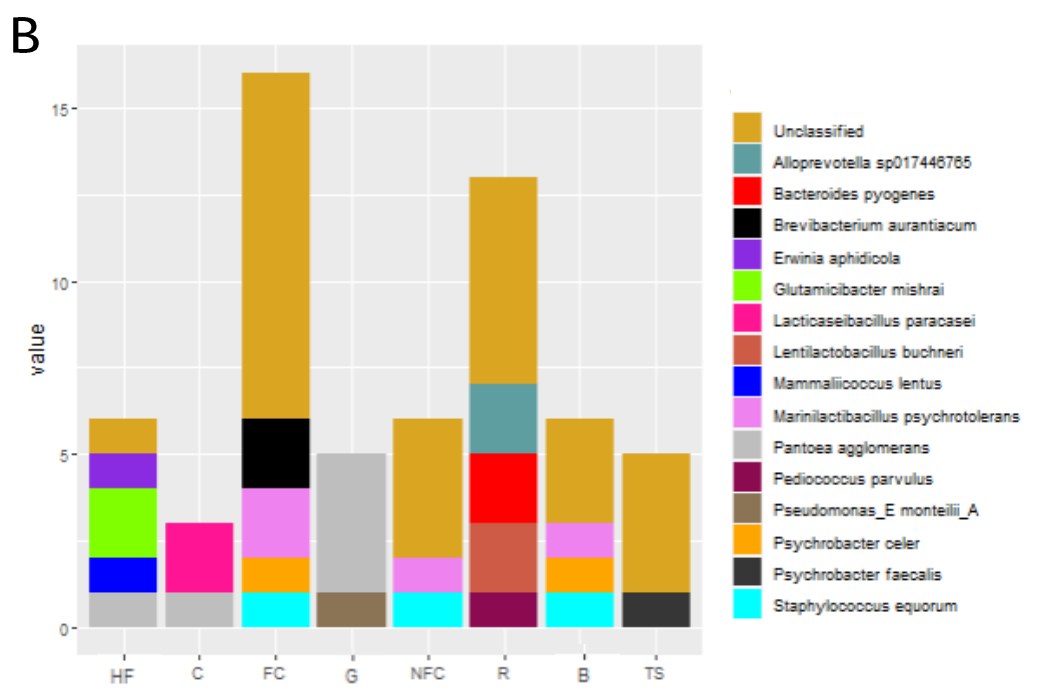**C**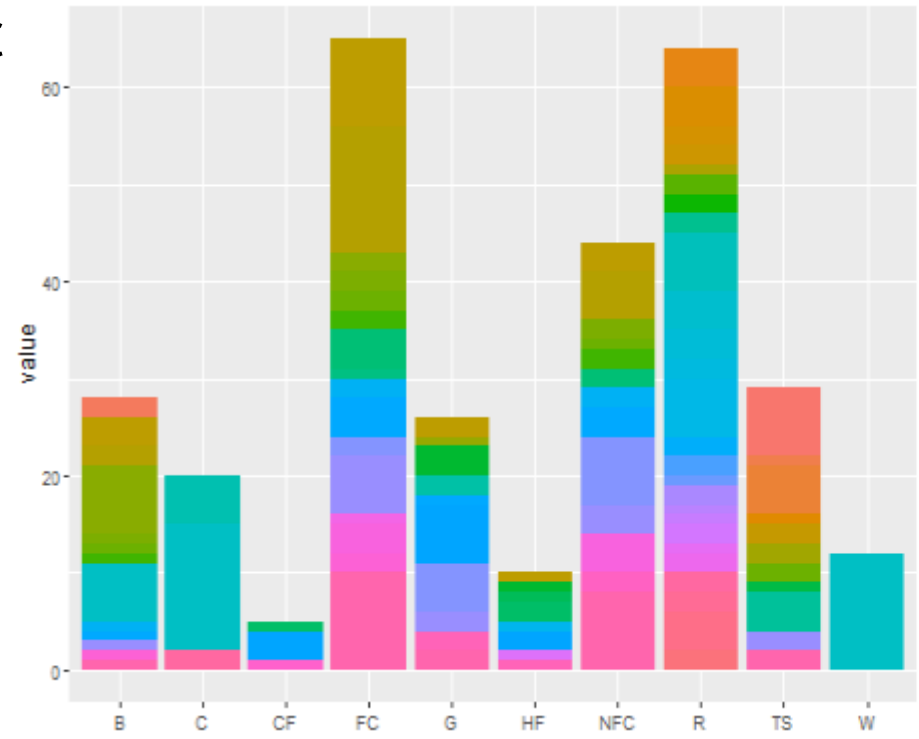**D**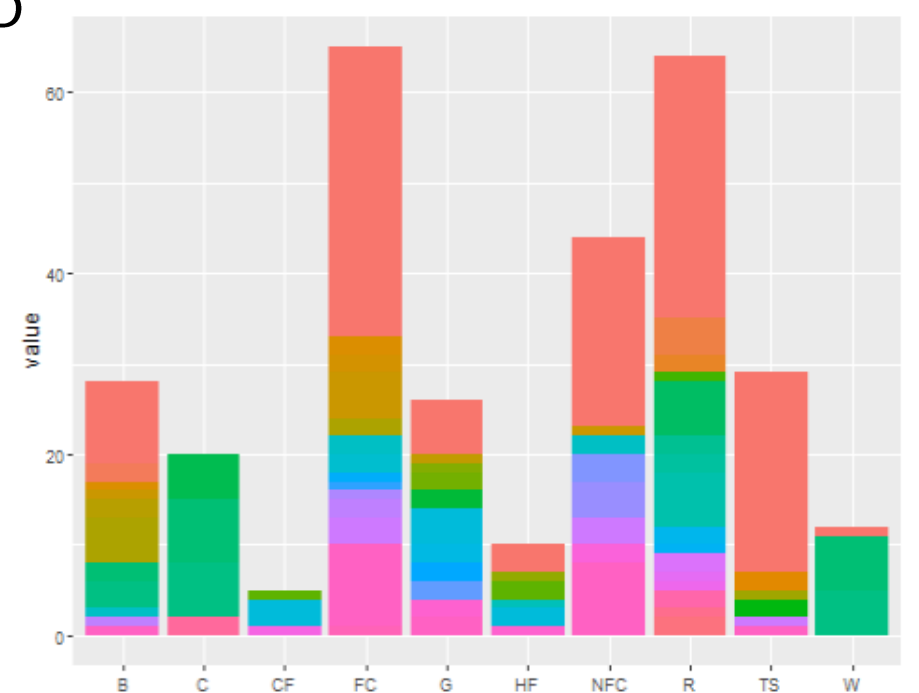

Supplement: Supplementary file 17 — Additional file 16: Supplementary Figure S6. Bar chart representation of the obtained high quality MAGs at genera (A) and species (B) levels, and medium-quality MAGs at genera (C) and species (D) taxonomic ranks. [file 40168_2024_1980_MOESM16_ESM.pdf]
