# Supplementary material for: Shotgun metagenomic sequencing reveals the influence of artisanal dairy environments on the microbiomes, quality, and safety of Idiazabal, a raw ewe milk PDO cheese
Source: Microbiome. 2024 Dec 20;12:262. doi: 10.1186/s40168-024-01980-0 (PMC11662609; doi:10.1186/s40168-024-01980-0)

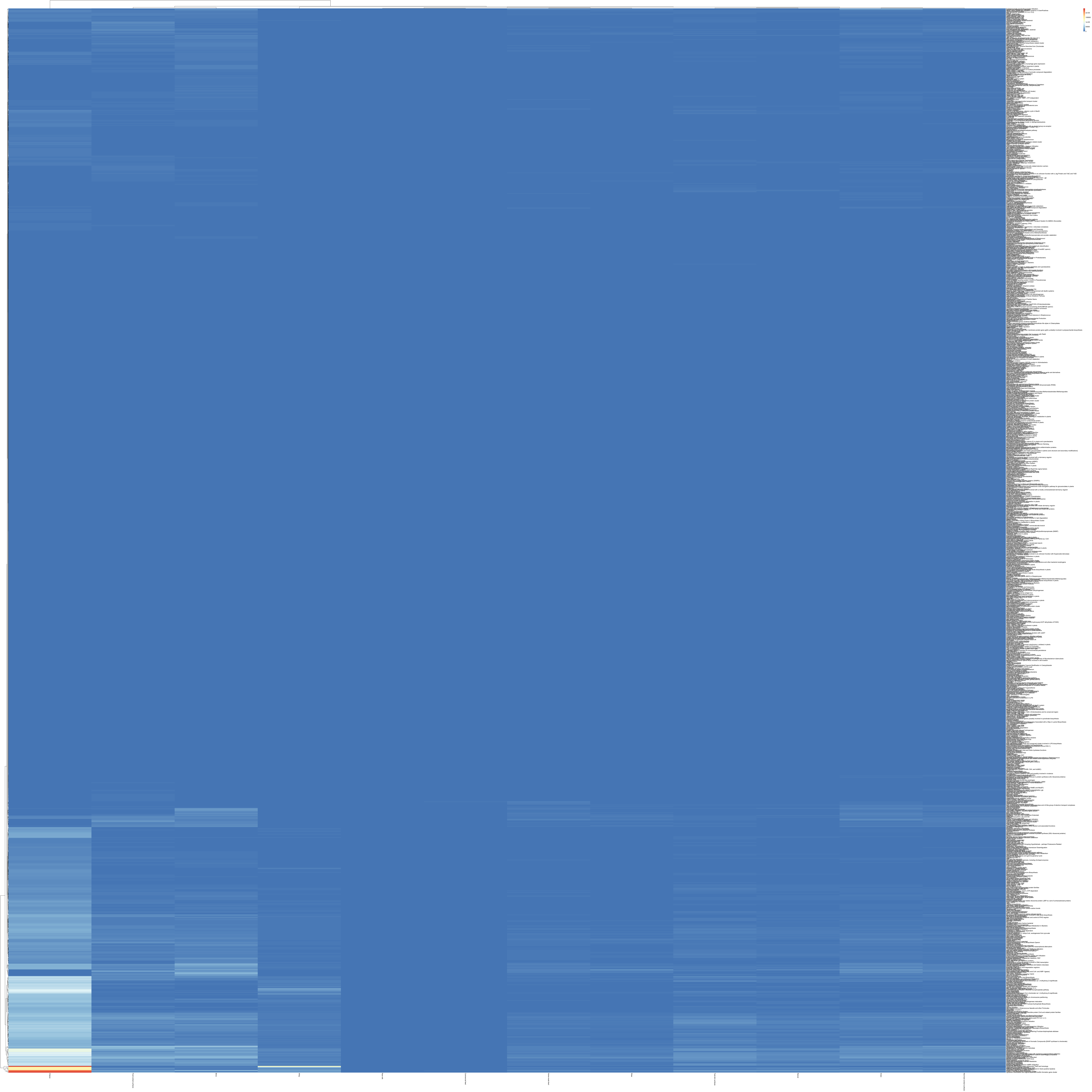

Supplement: Supplementary file 16 — Additional file 15: Supplementary Figure S5. Heatmap representation of metabolic pathways at subsystem level 3 according to sample type. [file 40168_2024_1980_MOESM15_ESM.pdf]
